# Evaluating kratom alkaloids using PHASE

**DOI:** 10.1371/journal.pone.0229646

**Published:** 2020-03-03

**Authors:** Christopher R. Ellis, Rebecca Racz, Naomi L. Kruhlak, Marlene T. Kim, Alexey V. Zakharov, Noel Southall, Edward G. Hawkins, Keith Burkhart, David G. Strauss, Lidiya Stavitskaya

**Affiliations:** 1 Division of Applied Regulatory Science, Office of Clinical Pharmacology, Office of Translational Sciences, Center for Drug Evaluation and Research, United States Food and Drug Administration, Silver Spring, Maryland, United States of America; 2 National Center for Advancing Translational Sciences, National Institutes of Health, Rockville, Maryland, United States of America; 3 Controlled Substances Staff, Center for Drug Evaluation and Research, United States Food and Drug Administration, Silver Spring, Maryland, United States of America; University of Parma, ITALY

## Abstract

Kratom is a botanical substance that is marketed and promoted in the US for pharmaceutical opioid indications despite having no US Food and Drug Administration approved uses. Kratom contains over forty alkaloids including two partial agonists at the mu opioid receptor, mitragynine and 7-hydroxymitragynine, that have been subjected to the FDA’s scientific and medical evaluation. However, pharmacological and toxicological data for the remaining alkaloids are limited. Therefore, we applied the Public Health Assessment via Structural Evaluation (PHASE) protocol to generate *in silico* binding profiles for 25 kratom alkaloids to facilitate the risk evaluation of kratom. PHASE demonstrates that kratom alkaloids share structural features with controlled opioids, indicates that several alkaloids bind to the opioid, adrenergic, and serotonin receptors, and suggests that mitragynine and 7-hydroxymitragynine are the strongest binders at the mu opioid receptor. Subsequently, the *in silico* binding profiles of a subset of the alkaloids were experimentally verified at the opioid, adrenergic, and serotonin receptors using radioligand binding assays. The verified binding profiles demonstrate the ability of PHASE to identify potential safety signals and provide a tool for prioritizing experimental evaluation of high-risk compounds.

## Introduction

The Center for Drug Evaluation and Research at the US Food and Drug Administration recently developed the Public Health Assessment via Structural Evaluation (PHASE) protocol as a tool for characterizing newly identified substances that lack *in vitro* and *in vivo* data[1]. PHASE, originally developed for characterizing fentanyl analogs, is a computational-based approach for assessing the risk a drug or compound may pose to public safety. The methodology has broad applicability and can rapidly generate *in silico* binding profiles of any drug or compound. PHASE is comprised of three components that: 1) quantify the structural similarity of a compound to other controlled substances, 2) predict the likely biological targets of a compound, and 3) estimate the binding affinity of a compound at the receptor(s) of interest. Comprehensively testing all suspect compounds against all potential biological targets would be prohibitively expensive and time-consuming. Instead, *in silico* binding profiles generated by PHASE can prioritize compounds of concern and direct which biological targets the compounds should be initially tested against *in vitro*. This is particularly useful for evaluating large sets of compounds that lack significant *in vitro* or *in vivo* data such as the specific indole and oxindole alkaloid structures found in kratom.

Kratom is a botanical substance derived from the leaves of *Mitragyna speciosa* that exhibits dose-dependent effects when administered. Low doses of kratom produce stimulatory effects, while larger doses are associated with opioid-like sedative effects[2]. Traditionally, kratom is consumed as a tea or by chewing the plant leaves to alleviate mental and physical fatigue[3]. More recently, however, recreational use of kratom has become popular in the EU and US to produce a ‘legal high’ which has led to a growing number of calls to poison control centers[4, 5]. Furthermore, kratom is being used for pharmaceutical opioid indications including pain management, mood disorders, and alleviating opioid withdrawal symptoms[6]. This is particularly concerning as there are no published clinical studies that describe the results of kratom administration for any of these therapeutic purposes. While animal models have indicated that the main component of kratom, mitragynine, has a low abuse potential [7, 8], mitragynine may be metabolized into 7-hydroxymitragynine [9], a component of kratom with demonstrated abuse potential and drug dependency[10, 11], exhibited analgesic effects that exceed the potency of morphine [12], and is a suspected adulterant of commercial kratom products [13].

Kratom pharmacology is complex. Over 40 alkaloids have been identified in kratom leaves, and the concentrations vary depending on numerous factors including the region the plant is grown in, timing of its harvest, and the age of the plant [5, 14, 15]. Furthermore, the traditionally-prepared kratom tea appears to provide greater systemic exposure of mitragynine, the most abundant alkaloid of kratom [16]. Mitragynine and 7-hydroxymitragynine, two partial mu opioid receptor agonists present in kratom [17], have been subjected to the FDA’s medical and safety evaluation (Eight-Factor Analysis). However, pharmacological and toxicological data for the remaining alkaloids are limited, and the interpretation of experimental results is complicated by conflicting binding and functional assay results across species. For example, bioluminescence resonance energy transfer (BRET) functional assays [18, 19] demonstrated that mitragynine is a partial agonist at the human mu opioid receptor, while paynantheine is an antagonist at the human mu opioid receptor. In contrast, the same assays at rodent mu opioid receptors demonstrated that mitragynine is a competitive antagonist and paynantheine has no agonist or antagonist activity [17]. These mixed results across species create challenges when translating *in vitro* and *in vivo* results to the assessment of potential effects on human health[20].

**Fig 1:**
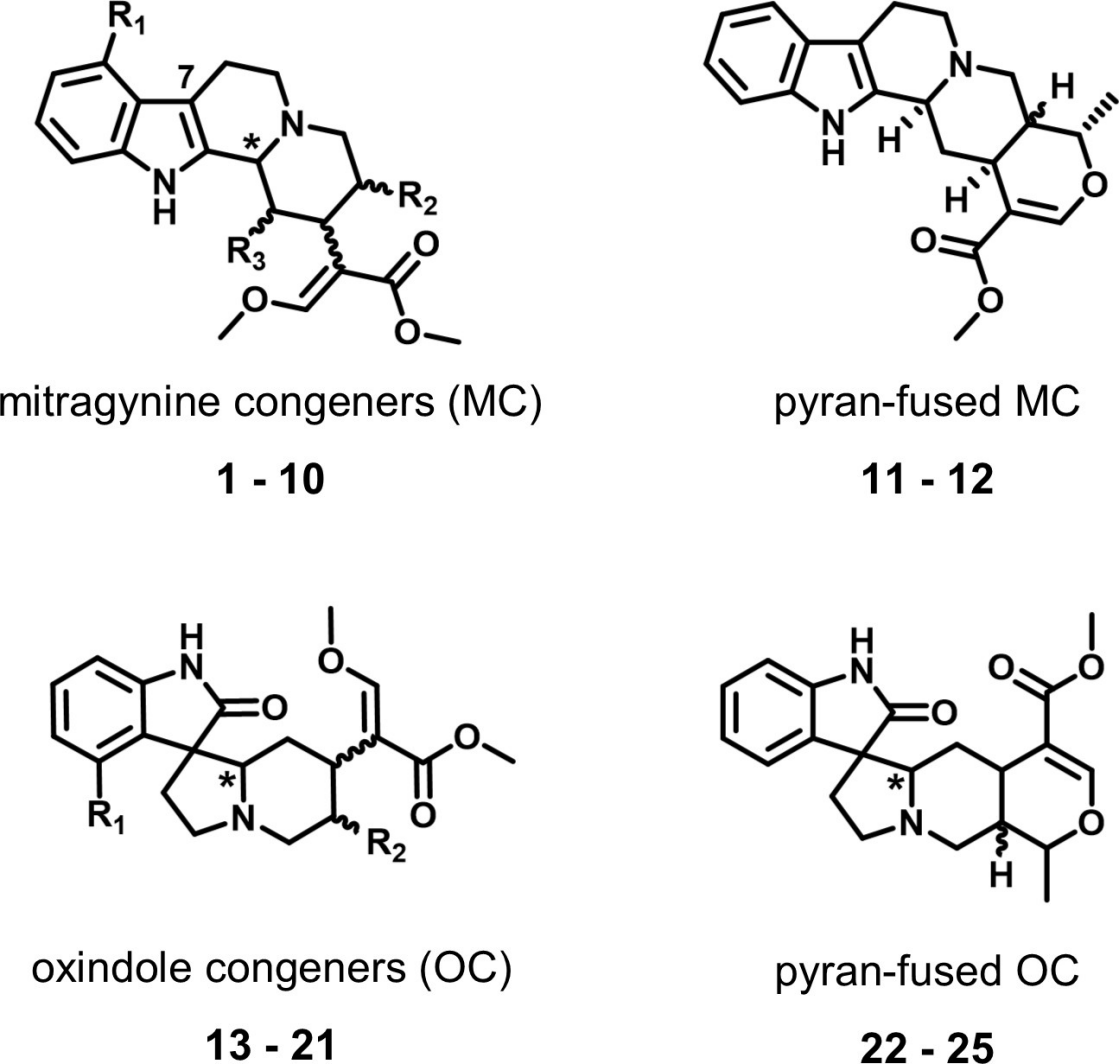
Alkaloids classes. The four general alkaloid classes include the mitragynine congeners (**1–10**), pyran-fused mitragynine congeners (**11–12**), oxindole congeners (**13–21**), and the pyran-fused oxindole congeners (**22–25**). The chiral centers that are addressed in this manuscript are indicated by wavy bonds and asterisks (*), and the locations of varying side chains are marked as R_1_, R_2_ and R_3_. A complete description of the structure and stereochemistry of alkaloids is presented in the supporting information.

In the current study, 25 kratom alkaloids were evaluated using PHASE and then a subset of commercially available kratom alkaloids were further evaluated using *in vitro* binding assays. The 25 alkaloids can be assigned to four general structural classes based on their underlying scaffold (Fig 1):

1. The mitragynine congeners (**1–10**) include mitragynine (**1**) and its stereoisomers speciogynine, speciociliatine, and mitraciliatine (**2–4**) as well as compounds with minor structural modifications (**8–10**) including the mu receptor partial agonist 7-hydroxymitragynine (**7**).
2. The pyran-fused mitragynine congeners, ajmalicine (**11**) and tetrahydroalstonine (**12**), have an additional ring in the core structure with respect to the mitragynine congeners.
3. The oxindole congeners (**13–21**) formed by a rearrangement product of the mitragynine congeners.
4. The pyran-fused oxindole congeners (**22–25**) have an additional ring in the core structure with respect to the oxindole congeners.

The mitragynine congeners account for roughly 85% of the total alkaloid concentration of kratom, and the remaining alkaloids each account for less than one percent of the total alkaloid concentration[5, 21]. The PHASE binding profiles of the kratom alkaloids indicated several biological targets associated with potential adverse effects. Therefore, the binding predictions were experimentally determined using radioligand binding assays for a subset of the kratom alkaloids to validate the utility of rapidly generating *in silico* binding profiles for compounds that lack significant experimental data to direct experimental inquiry. Furthermore, the prediction results and their validation demonstrated the broader utility of the PHASE approach to classes of compounds beyond fentanyl derivatives.

## Methods

### Structural similarity analysis

Chemical structures of the alkaloids and the controlled substances were downloaded from PubChem [22] and prepared using the ‘Quickprep’ function in Molecular Operating Environment (MOE) [23]. The Quickprep function performs an energy minimization and sets the protonation state of any titratable residues. The structural similarity of the alkaloids with respect to mitragynine and the controlled substances database was calculated using the Tanimoto equation for pairs of molecular fingerprints Structure fingerprints were generated using the MACCS166 Structural Keys[24]. The MACCS166 Structural Keys interrogate chemical structure using a series of 166 two-dimensional descriptors that account for the presence of common chemical features including charge, halogens, sulfur atoms, aromatic rings, connectivity, etc. The response to each of the 166 descriptor inquiries is stored in an array (fingerprint) that contains either a 1 or 0 depending on presence or absence of each descriptor, respectively. Once an array for each chemical structure has been constructed, the structural similarity of two compounds is calculated using a Tanimoto equation [25]. The Tanimoto coefficient quantifies the similarity between two compounds as the ratio of shared chemical features divided by the union of chemical features for each pair. Tanimoto coefficients range from 0 to 1, where compounds with a high degree of structural similarity have a Tanimoto coefficient near one, while structurally divergent compounds have a score near zero.

### Target binding predictions

Two software programs, SEAware by SEAChange Pharmaceuticals [26, 27] and Clarity by Chemotargets[28], were used to predict potential binding targets of the 25 kratom alkaloids. Both platforms use two-dimensional chemical structure to predict potential binding targets. This manuscript focuses on the opioid (mu, kappa, and delta), adrenergic (alpha-2A, 2B, and 2C), and serotonin receptors (5HT1A and 5HT2A). Predictive performance of these receptors has been previously assessed by their respective software developers [26, 27, 29]. Additional review of the training set compounds used to generate a prediction was completed to determine the relevance of a prediction.

SEAware predicts binding at approximately 2,300 targets throughout the body[26, 27]. SEAware compares the chemical structure of the drug of interest to the structures of other drugs and chemicals that are known to bind to each of these targets. Each target training set is generated from experimental binding data contained in ChEMBL version 22. A prediction and associated confidence score is generated for each target based on the structural similarity between the drug of interest to known binders. For this analysis, drug-target associations with a predicted binding affinity of less than 10 μM are considered a positive prediction of binding.

Clarity v2.1 predicts binding and associated activity (*i*.*e*., agonism and antagonism) at 3,791 targets throughout the body. Clarity compares the chemical structure of the drug of interest to the structures of other drugs and chemicals that are known to bind to each of these targets using six independent approaches, including machine learning, cross pharmacology indices, and simplest active subgraph[28]. The training set is generated from experimental binding data contained in ChEMBL version 24, patents, literature, and other public databases. A prediction and associated confidence score is generated for each target along with potential activity and binding affinity. For this analysis, predictions with a confidence score greater than 0.3 and a predicted K_i_ of less than 10 μM are considered a positive prediction of binding.

### Molecular docking

Complete details of the mu opioid receptor molecular docking model, the associated regression model used for the binding affinity, and validation experiments are published separately [1, 30]. In brief, the mu opioid receptor, crystallized in the active state with the agonist BU72 occupying the binding site, was used for the starting configuration for molecular docking (PDBID: 5C1M)[31]. The crystal structure was prepared using the `QuickPrep' function within MOE [23]. `QuickPrep' sets the protonation states of all titratable residues within the protein structure and performs an energy minimization to remove steric clashes. All water molecules in the crystal structure were maintained in the structure preparation and included during docking and pose evaluation.

The alkaloids that were predicted to bind the mu opioid receptor by either SEAware or Clarity were further evaluated using molecular docking. Each alkaloid was docked using the Triangle Matcher placement algorithm and refined using the induced fit protocol. No pharmacophore was used for the placement of the alkaloids into the mu opioid receptor active site. The protein and alkaloids were modeled with the Amber force field in combination with the Extended Hückel Theory parameterization for small molecules [32–34]. The docked alkaloids were scored with the Generalized-Born Volume Integral/Weighted Surface area (GBVI/WSA) scoring function[35]. For each alkaloid, the molecular docking procedure generates a lowest energy conformation (strongest binding) of the alkaloid in complex with the mu opioid receptor. The strength of the binding interaction is estimated using an empirical scoring function that quantifies the non-covalent interactions between the alkaloid and the binding pocket to provide a binding energy (Score). Alkaloids that receive lower binding scores with the mu opioid receptor are predicted to have stronger binding affinities.

### Binding assay protocol

Mitragynine (**1**), speciogynine (**2**), 7-hydroxymitragynine (**7**), ajmalicine (**11**), tetrahydroalstonine (**12**), corynoxine B (**14**), isorhynchophylline (**16**), corynoxeine (**20**), isocorynoxeine (**21**) were selected due to their availability in the National Center for Advancing Translation Science (NCATS) compound library and tested for binding at the opioid receptors (mu, kappa, delta), adrenergic receptors (alpha-2A, 2B, 2C), and serotonin receptors (5HT1A and 5HT2A) by the National Institute of Mental Health Psychoactive Drug Screening Program (NIMH PDSP)[36]. In brief, the alkaloids were tested for binding at the opioid, adrenergic, and serotonin receptors at a concentration of 10 μM in quadruplicate. Alkaloids with demonstrated binding at 10 μM underwent secondary concentration-response testing to determine the equilibrium binding affinity. The reported binding affinity of each compound is the average of two concentration-response binding assays. The radioligands and human cell lines used for making the membrane pellets for the binding assays vary between the receptors. The mu, kappa, and delta opioid receptors were derived from stable human HEK cells (ATCC CRL-11268; mycoplasma free), and [^3^H]-DAMGO, [^3^H]-U69593, and [^3^H]-DADLE were used as the displaced radioligand to measure the alkaloid binding affinity, respectively. The adrenergic alpha2A and alpha2C receptors were derived from stable MDCK cell line (ATCC CCL-34; mycoplasma free), while the alpha2B receptors were derived from transient HEKT cell lines. [^3^H]-Rauwolscine was used as the displaced radioligand for the three adrenergic receptors. Finally, the serotonin 5HT1A and 5HT2A receptors were derived from stable CHO parental cells (ATCC CCL-61; mycoplasma free) and transient HEKT cells (ATCC CRL-11268; mycoplasma free), respectively, and [^3^H]-8-OH-DPAT and [^3^H]-clozapine were used to measure the alkaloid binding affinity. Cell culture conditions, radioligands, concentrations, and conditions used for the opioid (mu, kappa, delta), adrenergic (alpha-2A, 2B, 2C), and serotonin (5HT1A and 5HT2A) receptor binding assays are listed as supporting information (S1 Data). Complete details of the binding assay conditions and protocols are provided in the NIMH PDSP Assay protocol book[37].

## Results and discussion

### Structural similarity analysis

The structural similarity of the alkaloids with respect to mitragynine and all currently scheduled drugs was first assessed using MACCS166 molecular fingerprints and the Tanimoto coefficient (T_c_) [25]. The MACCS166 molecular fingerprint does not capture any information about atom chirality. Therefore, the 25 alkaloids under consideration map to 12 distinct structures. The structural similarity assessment quantifies the degree of similarity between sets of compounds instead of relying on visual inspection.

### Structural similarity of the alkaloids with respect to mitragynine

The structural similarity score (T_c_) of the alkaloids with respect to mitragynine (**1**) ranges from 0.73 to 1.00. Speciogynine, speciociliatine, and mitraciliatine (**2–4**) are mitragynine stereoisomers, and therefore, map to the same two-dimensional structure (T_c_ = 1.00, Table 1). Paynantheine (**5**), corynantheidine (**6**), and 7-hydroxymitragynine (**7**) only differ from mitragynine by the addition or removal of a single chemical feature. Despite compounds **5–7** all differing by a single chemical feature, the similarity score of 7-hydroxymitragynine (T_c_ = 0.84) is lowered because the introduction of the hydroxyl group changes the chemical environment of the indole ring which increases the number of MACCS structural features associated with 7-hydroxymitragynine. In contrast, the introduction of an unsaturation (paynantheine, **5**) and removal of the methoxy group (corynantheidine, **6**) both decreased the total number of MACCS structural keys, while retaining the majority of the overlapping features with mitragynine (**1**). The remaining mitragynine congeners, mitragynaline (**8**), mitragynalinic acid (**9**), and corynantheidalinc acid (**10**) differ from mitragynine by the presence of an additional aldehyde sidechain at R3 and unsaturation in the core scaffold, however despite these differences, compounds (**8–10**) have substantial structural overlap with mitragynine (T_c_ = 0.84–0.89). Similarly, the pyran-fused mitragynine congeners (**11–12**) share numerous chemical features with mitragynine and have a high similarity score with respect to mitragynine (T_c_ = 0.92). Finally, the different chemical scaffold of the oxindole congeners and pyran-fused oxindole congeners (**13–25**) decreases the structural similarity but many features still overlap with mitragynine (T_c_ = 0.73–0.81).

**Table 1:** Structural similarity analysis of the kratom alkaloids with respect to (w.r.t) mitragynine and the controlled substance database. The MACCS166 molecular fingerprint only considers two-dimensional structure. Therefore, the 25 alkaloids under consideration map to 12 unique chemical structures.

ID	Name	Number of MACCS166 structural features	Similarity w.r.t. mitragynine, Tc (Overlapping features)	Most similar Controlled Substance	Structural similarity w.r.t. controlled substance, Tc (Overlapping features)
1–4	Mitragynine, Speciogynine, Speciociliatine, Mitraciliatine	63	1.00 (63)	Acetorphine	0.74 (55)
5	Paynantheine	60	0.92 (59)	Nicocodeine	0.71 (49)
6	Corynantheidine	58	0.92 (58)	Acetorphine	0.70 (51)
7	7-Hydroxymitragynine	71	0.84 (61)	Acetorphine	0.78 (60)
8	Mitragynaline	60	0.86 (57)	Nicocodeine	0.66 (47)
9	Mitragynalinic acid	58	0.89 (57)	Nicocodeine	0.71 (46)
10	Corynantheidalinic acid	55	0.84 (54)	Lysergic acid	0.71 (42)
11–12	Ajmalicine, Tetrahydroalstonine	57	0.88 (56)	Nicocodeine	0.72 (48)
13–16	Corynoxine, Corynoxine B, Rhynchophylline, Isorhynchophylline	67	0.81 (58)	Remifentanil	0.72 (52)
17–19	Speciofoline, Isospeciofoline, Mitrafoline	71	0.81 (60)	Acetorphine	0.76 (59)
20–21	Corynoxeine, Isocorynoxeine	63	0.73 (53)	Nicomorphine	0.66 (48)
22–25	Mitraphylline, Isomitraphylline, Speciophylline, Isospeciophylline	65	0.75 (55)	Thebacon	0.72 (52)

### Structural similarity of the alkaloids with respect to controlled substances

The controlled substance database contains all currently scheduled drugs and is linked to a manually-curated chemical structure [1]. Evaluating the chemical similarity of a compound with respect to controlled substances can provide insight into potential binding targets as compounds with a high degree of structural similarity may have similar pharmacological profiles. With the exception of corynantheidalinic acid (**10**), the remaining, 24 alkaloids are most structurally similar to controlled opioids (T_c_ ranging from 0.66 to 0.78). However, the kratom alkaloids have different chemical scaffolds than all currently controlled opioids.

Specifically, the mitragynine and pyran-fused mitragynine congeners (**1–9,11–12**) share the most structural features with the morphine analogs, acetorphine and nicocodeine (T_c_ = 0.70–0.78). Interestingly, the oxindole congeners were most structurally similar to two different classes of opioids. Compounds **13–16** were most similar to remifentanil, a fentanyl analog, while compounds **17–19** were again most similar to acetorphine, a morphine analog. Corynoxeine and isocorynoxeine (**20–21**) shared the most structural features with nicomorphine, however, the Tanimoto coefficient was low (T_c_ = 0.66) indicating that the two compounds do not share a substantial number of overlapping features. Interestingly, 7-hydroxymitragynine (**7**), a compound with a strong binding affinity at the mu opioid receptor[17], shared many structural features with the potent opioid acetorphine (T_c_ = 0.78).

### Binding target predictions

The biological targets of the 25 alkaloids were predicted using Clarity [28] and SEAware [26, 27]. Each prediction platform contains models for thousands of potential binding targets. Alkaloids that have experimentally determined binding targets are included in each of the model training sets. Given the complex nature of the alkaloid stereochemistry and the impact stereochemistry has on both binding affinity and function, the target prediction platforms were used as an initial characterization to categorize compounds as predicted binders (K_i_ < 10 μM) or predicted non-binders (K_i_ > 10 μM). These predictions can be used to guide more focused studies to evaluate experimental binding and function at the identified targets with abuse potential. For example, naloxone binds the mu opioid receptor, but as an antagonist it provides a tremendous public health benefit. However, we have limited the discussion to binding affinity to demonstrate the prediction platforms’ ability to generate binding profiles to guide experimental inquiry.

### Opioid receptors

The opioid receptors are the primary target of concern given the opioid-like properties of kratom. Compounds **1–4** and **6–7** are included in the mu, kappa, and delta opioids receptor model training sets within Chemotargets and SEAware (Table 2). Clarity and SEAware correctly predict that paynantheine (**5**) binds to the mu opioid receptor with sub 10 μM binding affinity, which is consistent with reported human and rodent mu opioid receptor binding affinity values of 0.41 ± 0.2 and 0.67 ± 0.08 μM, respectively [17]. However, neither software platform predicted paynantheine to bind to the kappa opioid receptor, where demonstrated human and rodent values of 2.6 ± 0.4 and 0.89 ± 0.3 μM, respectively, have been reported[17]. Clarity predicts that paynantheine binds to the delta opioid receptor with sub 10 μM, while SEAware does not. However, the binding affinity of paynantheine at human and rodent delta opioid receptors differs significantly. Paynantheine binds to the rodent delta opioid receptor with 4.3 ± 0.7 μM binding affinity, while no binding was measured at the human delta opioid receptor under 10 μM. Therefore, Clarity’s prediction for paynantheine binding to the delta opioid receptor with sub 10 μM binding affinity correctly reflects its behavior towards non-human receptors, which is reasonable given that its nearest neighbors in the training set have binding data from non-human receptors. Finally, mitragynaline (**8**), mitragynalinic acid (**9**), and corynantheidalinic acid (**10**) were not predicted to bind to any of the opioid receptors despite sharing many structural features with the known binder, mitragynine (T_c_ = 0.84–0.89).

**Table 2:** Alkaloid binding predictions at the opioid receptors. Compounds that were included in each model training set are reported as ‘T’, while compounds that are predicted to have a binding affinity > 10 μM are reported as ‘-‘. Clarity and SEAware use different metrics associated with probability of binding to respective drug targets. For positive predictions of binding, Clarity confidence scores and SEAWare p-values are provided.

Compound Identifiers		mu		kappa		delta	
ID	Name	Clarity	SEAware	Clarity	SEAware	Clarity	SEAware
1–4	Mitragynine, Speciogynine, Speciociliatine, Mitraciliatine	T	T	T	T	T	T
5	Paynantheine	0.78	1.0e-8	-	-	0.76	-
6	Corynantheidine	T	T	T	T	T	T
7	7-Hydroxymitragynine	T	T	T	T	T	T
8	Mitragynaline	-	-	-	-	-	-
9	Mitragynalinic acid	-	-	-	-	-	-
10	Corynantheidalinic acid	-	-	-	-	-	-
11–12	Ajmalicine, Tetrahydroalstonine	0.86	-	-	-	-	-
13–16	Corynoxine, Corynoxine B, Rhynchophylline, Isorhynchophylline	-	2.8e-13	0.80	-	-	-
17–19	Speciofoline, Isospeciofoline, Mitrafoline	0.37	1.5e-13	0.37	-	0.37	-
20–21	Corynoxeine, Isocorynoxeine	-	3.0e-10	-	-	0.37	-
22–25	Mitraphylline, Isomitraphylline, Speciophylline, Isospeciophylline	0.37	1.1e-6	0.37	-	0.37	-

The pyran-fused mitragynine congeners, ajmalicine (**11**) and tetrahydroalstonine (**12**) are predicted to bind the mu opioid receptor by Clarity, while neither platform predicts compounds **11–12** will bind the kappa and delta opioid receptors. The binding predictions of the oxindole mitragynine congeners (**13–21**) at the opioid receptors varied between platforms. Indeed, only three of the nine predictions are shared between both platforms. SEAware predicts that compounds **13–16** and compounds **20–21** bind the mu opioid receptor and Clarity does not, while Clarity predicts compounds **13–19** bind the kappa opioid receptor and SEAware does not. Similarly, the pyran-fused oxindoles compounds **22–25** have mixed predictions between the two platforms. SEAware only predicts **22–25** bind the mu opioid receptor, while Clarity predicts that compounds **22–25** bind the mu, kappa, and delta opioid receptors. It is worth noting that Clarity’s confidence of the binding prediction for compounds **17–25** is quite low (0.37). This result demonstrates the binding prediction platforms’ ability to discriminate between compounds that are highly structurally similar to compounds known to bind particular targets. Additionally, these results highlight the advantages of using multiple prediction platforms for detecting safety signals because the two platforms utilize different structural perspectives. Furthermore, consensus predictions between the target predication platforms provide increased confidence in the predictions.

### Adrenergic and serotonin receptors

The kratom alkaloids interact with additional cellular targets and are not limited to the opioid receptors. Indeed, reports have shown mitragynine binds to several non-opioid receptors, including adrenergic receptors and cardiac potassium channels [38, 39]. However, determining the probable binding partners for a set of compounds is challenging considering that there are thousands of potential binding targets. Therefore, the target prediction platforms provide a valuable tool for guiding where to begin experimental verification. In addition to the opioid receptors discussed in the previous section, the target prediction platforms indicated the adrenergic (Alpha-2A, -2B and -2C) and serotonin (5-HT1A and 5-HT2A) receptors as likely binding targets for the mitragynine congeners **1–7** and the pyran-fused mitragynine congeners **11–12** (Table 3). The adrenergic and serotonin receptors were selected as the secondary targets of concern because the adrenergic alpha-2 receptors have effects on heart rate and blood pressure[40] and the serotonin receptors are associated with psychological functions such as aggression, anxiety, and depression[41]. In addition, serotonin neurotransmission has a complex role in seizure pathogenesis[42].

**Table 3:** Alkaloid binding predictions at the adrenergic and serotonin receptors. Compounds that were included in each model training set are marked ‘T’, while compounds that are predicted to have a binding affinity > 10 μM are marked ‘-‘. Clarity and SEAware use different metrics associated with probability of binding to the respective drug targets. For positive predictions of binding, Clarity confidence scores and SEAWare p-values are provided.

		Adrenergic Receptors						Serotonin Receptors			
		Alpha-2A		Alpha-2B		Alpha-2C		5-HT1A		5-HT2A	
ID	Name	Clarity	SEAware	Clarity	SEAware	Clarity	SEAware	Clarity	SEAware	Clarity	SEAware
1–4	Mitragynine, Speciogynine, Speciociliatine, Mitraciliatine	0.92	4.0e-15	0.85	8.4e-9	-	5.1e-13	-	-	0.54	-
5	Paynantheine	0.73	1.2e-14	0.73	8.4e-9	0.85	7.1e-13	-	-	0.79	-
6	Corynantheidine	0.92	2.8e-20	0.92	1.1e-11	-	2.7e-17	0.56	-	0.76	-
7	7-Hydroxymitragynine	0.31	-	0.31	-	0.32	-	-	-	-	-
8	Mitragynaline	-	-	-	-	-	-	-	-	-	-
9	Mitragynalinic acid	-	-	-	-	-	-	-	-	-	-
10	corynantheidalinic acid	-	2.3e-15	-	2.0e-8	-	1.0e-12	-	-	-	-
11–12	Ajmalicine, Tetrahydroalstonine	T	T	T	T	-	-	0.32	-	-	-
13–16	Corynoxine, Corynoxine B, Rhynchophylline, Isorhynchophylline	-	-	-	-	-	-	-	-	-	-
17–19	Speciofoline, Isospeciofoline, Mitrafoline	-	-	-	-	-	-	-	-	-	-
20–21	Corynoxeine, Isocorynoxeine	-	-	-	-	-	-	-	-	-	-
22–25	Mitraphylline, Isomitraphylline, Speciophylline, Isospeciophylline	-	1.4e-7	0.64	-	-	-	0.54	-	-	-

### Evaluating key binding interactions at the mu opioid receptor

**Fig 2:**
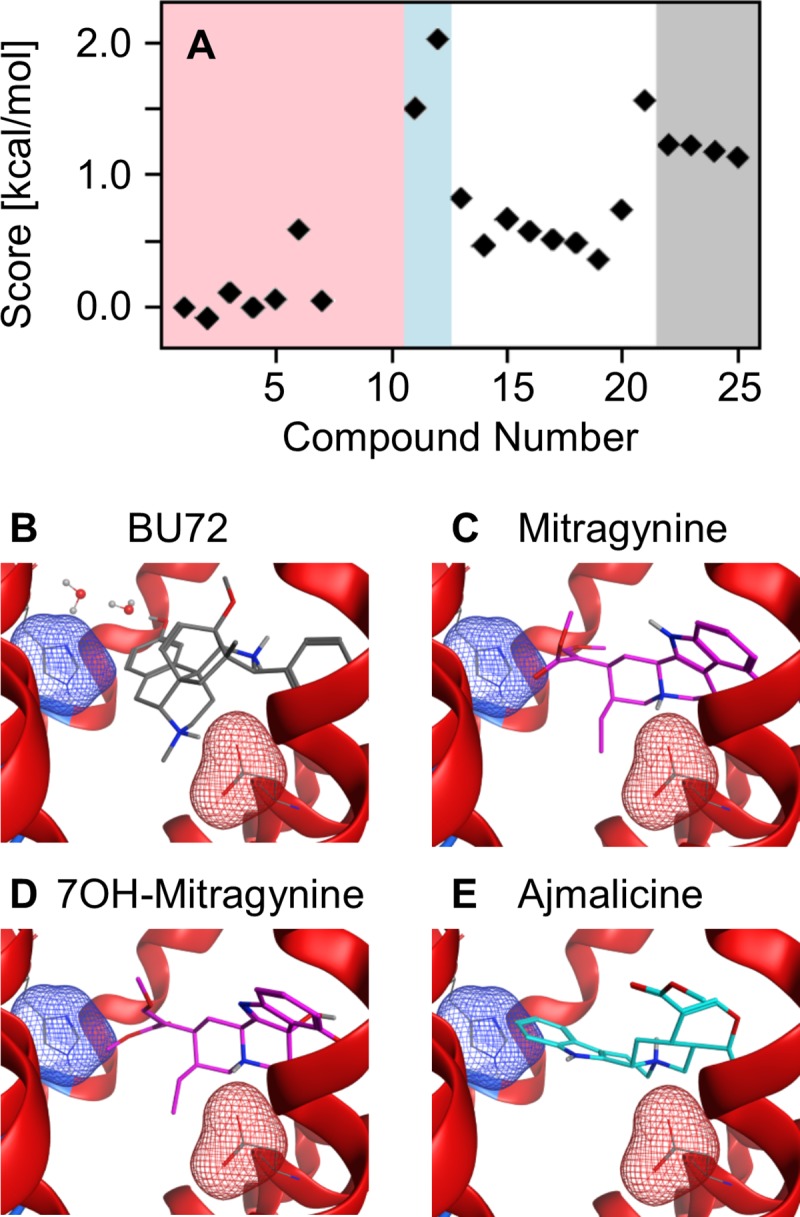
Molecular docking evaluation of the alkaloids at the mu opioid receptor. (A) The docking score of the mitragynine congeners (red), pyran-fused mitragynine congeners (blue), oxindole congeners (white), and the pyran-fused oxindole congeners (gray) normalized to mitragynine. (B) Crystal structure of the mu opioid receptor in complex with the agonist, BU72. (C-E) Lowest energy docking configuration of mitragynine (**1**), 7-hydroxymitragynine (**7**), and ajmalicine (**11**) with the mu opioid receptor. In panels B-E, the negatively charged sidechain of Asp147 that forms the salt bridge with the positively charged amine of each opioid is highlighted by the red mesh surface, and the aromatic sidechain of His297 is represented by the blue mesh surface.

Methodologies that rely on two-dimensional chemical structure cannot account for differences in binding affinity and function of the alkaloid stereoisomers [17]. Therefore, considering the three-dimensional structure of both the chemical and biological target is advantageous. The mu opioid molecular docking model was used to provide an atomically-detailed description of the interactions between the alkaloids and the binding pocket of the mu opioid receptor [30]. The twenty-two alkaloids predicted to bind the mu opioid receptor by either target prediction platform were docked with the mu opioid receptor[30]. The docking scores of the twenty-two alkaloids were shifted to set the docking score of mitragynine (**1**) to zero (Fig 2). Compounds with a higher docking score than mitragynine suggest a compound is less likely to bind the mu opioid receptor, while compounds with a more negative score indicate a higher likelihood of binding the mu opioid receptor. Little variation was observed in the docking scores among the mitragynine congeners (**1–7**, shaded red). With the exception of corynantheidine (**6**), the docking score of the mitragynine congeners only varied by 0.1 kcal/mol. However, the pyran-fused mitragynine congeners, ajmalicine (**11**) and tetrahydroalstonine (**12**), have a molecular docking score of 1.5 and 2.0 kcal/mol higher than mitragynine, respectively. The differences in the binding scores of the mitragynine and pyran-fused mitragynine congeners is significant for two reasons: 1) It demonstrates the scoring function is sensitive to stereochemistry, and 2) it indicates that despite both mitragynine and ajmalicine being predicted to bind the mu opioid receptor, it is likely that mitragynine congeners have stronger interactions with the mu opioid receptor. The oxindole (**13–21**) and pyran-fused oxindole congeners (**22–25**) also have higher binding scores than mitragynine. The increased docking score of the alkaloids with respect to mitragynine and 7-hydroxymitragynine is consistent with the structure-activity relationship of the mitragynine scaffold which is sensitive to structural modification[20].

The docking poses of the alkaloids within the mu opioid receptor binding pocket provide insight into the weaker binding affinity of mitragynine as compared to morphine and fentanyl analogs. Key interactions between morphine analogs and the mu opioid receptor include a salt bridge between the positively charged amine and a negatively charged aspartic acid (Asp147) and a water-mediated hydrogen bonding interaction between the phenolic hydroxyl with a histidine (His297) in the binding pocket. These interactions are evident in the mu opioid receptor crystal structure complex with the morphinan agonist BU72[31] (Fig 2B) as well as the morphinan antagonist β-funalaltrexamine [43]. Similarly, a molecular docking study of the fentanyl analogs with the mu opioid receptor demonstrated the amine-aspartic acid salt bridge and direct aromatic stacking with His297 are critical for strong binding [30]. In contrast, mitragynine (**1**) and 7-hydroxymitragynine (**7**) adopt a unique pose within the mu opioid active site (Fig 2C and 2D) in which the salt bridge with Asp147 is maintained but no water-mediated hydrogen bonding interaction or direct aromatic stacking interaction with His297 occurs. Instead, the enol ether domain of mitragynine and 7-hydroxymitragynine is directed towards His297, which is consistent with a previous mitragynine docking study using AutoDock [17, 44]. The lowest energy configurations of the remaining mitragynine congeners (**2–6**) adopt similar poses to mitragynine and 7-hydroxymitragynine in that the enol ether is directed toward His297.

In contrast, the pyran-fused mitragynine congener ajmalicine (**11**) adopts a different conformation in the active site. Instead of directing the fused-pyran ring toward His297, the molecule flips and directs the aromatic ring towards His297, which weakens the salt bridge with Asp147 and increases the docking score of ajmalicine (Fig 2E). Interestingly, the lowest energy configurations of tetrahydroalstonine (**12**) directs the pyran-fused ring towards His297 similar to the mitragynine congeners. The lowest energy poses of the oxindole congeners varied considerably and the differences in stereochemistry significantly altered the poses between stereoisomers. Corynoxine (**13**) maintained the salt bridge with Asp147 while directing the indoxyl-like ring towards His297, while the lowest energy configurations of corynoxine B (**14**) did not maintain the salt bridge with Asp147 but still directed the indoxyl-like ring towards His297. Similarity, the lowest energy poses of rhynchophylline (**15**) and isorhynchophylline (**16**) were distinct. These results further demonstrate the necessity of complimentary methodologies that incorporate three-dimensional chemical structure.

### Evaluation of the target prediction platforms at the opioid, adrenergic and serotonin receptors

The binding affinity of a subset of the alkaloids (**1, 2, 7, 11, 12, 14, 16, 20, 21**) was evaluated at the opioid (mu, kappa, delta), adrenergic (alpha-2A,B,C), and the serotonin (5HT1A,B) receptors using radioligand binding assays at the National Institute of Mental Health Psychoactive Drug Screening Program (NIMH PDSP)[36]. The compounds were acquired from the National Center for Advancing Translational Science (NCATS) compound library.

### Opioid receptors

The binding affinity of mitragynine (**1**), speciogynine (**2**), and 7-hydroxymitragynine (**7**) was measured at the mu, kappa, and delta human opioid receptors (Table 4). These compounds were present in the target prediction model training sets with sub 10 μM binding affinity. Therefore, it was surprising that speciogynine did not have a measured binding affinity more potent than 10 μM. However, there are two explanations for the discrepancy: 1) mitragynine, a known binder in the training set, and speciogynine have the same two-dimensional chemical structure, and therefore, are treated identically by each software platform and 2) the measurement was taken at the human opioid receptor and the models do not make species-specific predictions. In fact, speciogynine has a binding affinity more potent than 10 μM at the mouse opioid receptor[17].

**Table 4:** Evaluation of the opioid receptor binding predictions. Compounds that were included in the model training sets are marked as ‘T’, compounds predicted to have sub 10 μM binding affinity are marked as ‘+’, and compounds predicted to have > 10 μM binding affinity are marked as ‘-‘. The predictions from Clarity and SEAware are presented on the left and right, respectively. Cells that are shaded green indicate that the two software predictions are in agreement and correct. Cells that are shaded red indicate that the two software predictions are in agreement and incorrect. Finally, cells that are not shaded indicate that the two software platforms have conflicting predictions.

		Mu		Kappa		Delta	
ID	Name	K_i_ [μM]	Prediction (Clarity/SEA)	K_i_	Prediction	K_i_	Prediction
1	Mitragynine	0.74	T T	1.3	T T	6.5	T T
2	Speciogynine	1.0	T T	3.6	T T	>10	T T
7	7-hydroxymitragynine	0.070	T T	0.32	T T	0.47	T T
11	Ajmalicine	8.96	+ -	>10	- -	>10	- -
12	Tetrahydroalstonine	>10	+ -	>10	- -	>10	- -
14	Corynoxine B	1.6	- +	>10	- +	7.6	- -
16	Isorhynchophylline	0.54	- +	>10	- +	6.4	- -
20	Corynoxeine	>10	- +	>10	- -	>10	+ -
21	Isocorynoxeine	>10	- +	>10	- -	>10	+ -

Although the conservative “1+ rule,” by which any single positive prediction from the two software programs yields a positive overall, is typically applied when predicting unknown chemicals using Clarity and SEAware, predictions from each individual software have been reported in Tables 4 and 5 due to the small sample size. In general, application of the “1+ rule” when combining predictions from two different models results in higher sensitivity and negative predictivity. As expected, an increase in false positive rate is also observed; however, this effect can be mitigated to some degree through the application of expert review. Yet, more importantly, the overall decrease in the false negative rate supports the regulatory imperative to protect public safety. Both platforms correctly predicted that the pyran-fused mitragynine congeners **11–12** would not bind to the kappa and delta opioid receptors. However, the predictions were mixed at the mu opioid receptor. Clarity predicted that ajmalicine (**11**) and tetrahydroalstonine (**12**) would bind to the mu opioid receptor, while SEAware did not. In this case, due to the use of two-dimensional chemical structure, neither platform could be entirely correct. Ajmalicine (**11**) and tetrahydroalstonine (**12**) have the same two-dimensional structure and, therefore, the same target predictions. However, ajmaline was found to have only a weak binding affinity (K_i_ = 8.96 μM), while the binding affinity of tetrahydroalstonine was greater than 10 μM, suggesting that their affinities may not be significantly different. This measurement is consistent with docking model that suggested the binding affinity of the pyran-fused mitragynine congeners is weaker with respect to mitragynine.

**Table 5:** Evaluation of the adrenergic and serotonin receptor binding predictions. Compounds that were included in the model training sets are marked as ‘T’, compounds predicted to have sub 10 μM binding affinity are marked as ‘+’, and compounds predicted to have > 10 μM binding affinity are marked as ‘-‘. The predictions from Clarity and SEAware are presented on the left and right, respectively. Cells that are shaded green indicate that the two software predictions agree and are correct. Cells that are shaded red indicate that the two software predictions are in agreement and incorrect. Finally, cells that are not shaded indicate that the two software platforms have conflicting predictions.

		Adrenergic Receptors						Serotonin Receptors			
		Alpha-2A		Alpha-2B		Alpha-2C		5-HT1A		5-HT2A	
**ID**	**Name**	**K**_**i**_ **[**μ**M]**	**Prediction (Clarity/SEA)**	**K**_**i**_	**Prediction**	**K**_**i**_	**Prediction**	**K**_**i**_	**Prediction**	**K**_**i**_	**Prediction**
1	Mitragynine	2.3	+ +	4.9	+ +	3.5	- +	5.8	- -	7.3	+ -
2	Speciogynine	0.36	+ +	2.6	+ +	0.68	- +	0.54	- -	2.9	+ -
7	7-Hydroxy mitragynine	>10	+ -	>10	+ -	>10	+ -	>10	- -	>10	- -
11	Ajmalicine	0.045	T T	0.043	T T	0.065	- T	0.42	+ -	>10	- -
12	Tetrahydroalstonine	0.018	T T	0.040	T T	0.053	- T	0.38	+ -	2.6	- -
14	Corynoxine B	>10	- -	>10	- -	>10	- -	>10	- -	>10	- -
16	Isorhynchophylline	4.8	- -	>10	- -	>10	- -	>10	- -	>10	- -
20	Corynoxeine	>10	- -	8.4	- -	>10	- -	>10	- -	>10	- -
21	Isocorynoxeine	>10	- -	>10	- -	>10	- -	>10	- -	>10	- -

Similar to the pyran-fused mitragynine congeners, the platforms had mixed results for the oxindole congeners (**14, 16, 20, 21**) at the mu opioid receptor. SEAware correctly predicted the binding affinity of corynoxine B (**14**) and isorhynochophylline (**16**) as being under 10 μM, while Clarity did not. However, Clarity correctly predicted that corynoxeine (**20**) and isocorynoxeine (**21**) would have a binding affinity greater than 10 μM. It is not surprising that each platform is consistent in its characterization across the oxindole congeners given the significant amount of structural similarity across this class of compounds. Corynoxine B (**14**) and corynoxeine (**20**) only differ by one unsaturated bond. In fact, 62 of the 63 corynoxeine MACCS structural features overlap with corynoxine B (**14**, T_c_ = 0.91).

**Fig 3:**
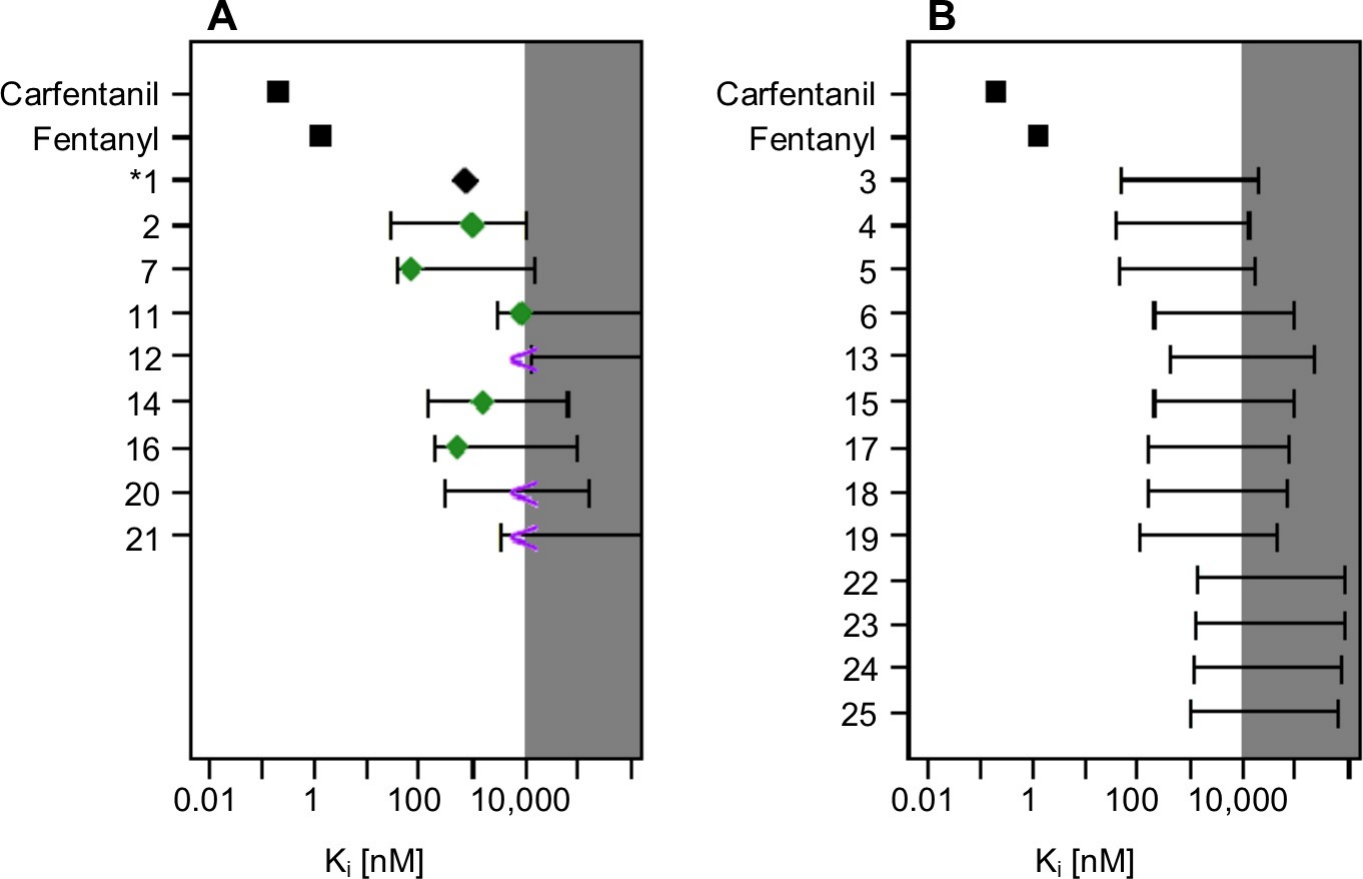
Binding affinity predictions using the mu opioid regression model. The model predictivity interval is shown by the error bars, while the experimentally determined binding affinities are presented as symbols in panel A. The measured binding affinities of fentanyl and carfentanil are presented, for comparison, as the black squares and the normalized mitragynine binding affinity is presented as the black diamond. Panel A also presents the measured binding affinities of the remaining eight kratom alkaloids. The green diamonds indicate the experimentally measured binding affinities, while the purple greater than symbol indicates that no binding occurred below 10 μM.

The molecular docking component of PHASE was used with a previously described mu opioid regression model to relate docking scores to binding affinity (Fig 3) [1, 30]. Despite recent advances, molecular docking models often fail to accurately relate docking scores to experimental observables (e.g. binding affinity) due to methodological limitations such as the lack of active site dynamics, poorly described solvent interactions, and a crude description of hydrogen bonding [45]. However, the mu opioid regression model was able to correctly relate key modifications of the fentanyl core to experimentally measured binding affinity with r = 0.86 for eight congeners [1]. These modifications included the addition of a methyl ester into the 4-anilido piperidine core (carfentanil) and removal of the N-phenethyl group (N-methyl fentanyl). The molecular docking/regression model also included compounds such as tramadol, propoxyphene, and methadone that do not contain the traditional fentanyl scaffold. In the regression model (details provided in the supporting information), a difference of 1.4 kcal/mol corresponds to an order of magnitude in binding affinity.

The generally applicability of the mu opioid regression model with a new scaffold was evaluated by comparing the predicted and measured binding affinity for the nine kratom alkaloids. The docking score of mitragynine (**1**) was set to its measured binding affinity (K_i_ = 740 nM) and the scores for the remaining alkaloids were calculated relative to mitragynine. The model predictivity interval spans approximately two orders of magnitude. However, it readily distinguishes between compounds with very strong binding affinity, e.g. carfentanil (K_i_ = 0.22 nM [46]) and fentanyl (K_i_ = 1.35 nM), from opioids with weaker binding affinity, e.g. tramadol (K_i_ = 12,500 nM). While functional assays are required to better understand the risk a particular compound may pose, relating the docking score to the binding affinity is useful for initial risk evaluation. Additionally, the predicted binding affinity provides a method for prioritizing experimental verification given a large influx of abused compounds that lack *in vitro* or *in vivo* data.

The docking procedure predicts that the mitragynine congeners will have the strongest binding affinity at the mu opioid receptor. Importantly, the experimentally determined binding affinity is within the range of the predicted binding affinity for the nine kratom alkaloids tested (green diamonds and purple greater than symbols, Fig 3A). The predicted binding affinity of the mitragynine congeners covers a broad range from approximately, 50–10,000 nM, suggesting that these alkaloids are unlikely to have sub nM binding affinity at the mu opioid receptor. The regression model accurately predicted the increased binding affinity of the pyran-fused mitragynine congeners (**11–12**). The lower bound of the binding affinity prediction for ajmalicine (**11**) and tetrahydroalstonine (**12**) was 3,100 nM and 13,000 nM, respectively, as compared to the measured values of 8,960 nM and >10,000 nM, respectively. Similarly, the lower bound of the binding affinity prediction of the pyran-fused oxindole congeners (**22–25**) is uniformly greater than 1,000 nM.

### Adrenergic and serotonin receptors

The binding affinity of kratom alkaloids at the mu opioid receptors has been previously reported[17, 47], and while interactions at other non-opioid central nervous system receptors have been reported, the binding affinity was not quantified[20, 38]. The binding prediction component of PHASE identified the adrenergic (Alpha-2A, B, C) and serotonin receptors (5-HT1A, 2A) as potential targets of the mitragynine congeners and pyran-fused mitragynine congeners. The binding predictions of mitragynine congeners (**1–7**) and the pyran-fused mitragynine congeners (**11–12**) highlight the challenges, but potential value, of using multiple binding prediction platforms for developing *in silico* binding profiles. The mitragynine congeners (**1**–**2**) and pyran-fused mitragynine congeners (**11–12**), have known sub 10 μM binding affinity at the adrenergic and serotonin receptors. Only Clarity predicted these compounds would bind to the serotonin 5HT2A receptor, while only SEAware predicted the mitragynine congeners would bind to the adrenergic-2C receptor. The two platforms show complementarity in their predictions by increasing the potential for identifying safety signals that require further experimental verification. Finally, both platforms were largely correct in predicting that the oxindole congeners (**14,16, 20, 21**) would not bind to the adrenergic or the serotonin receptors. Only isorhynchophylline (**16**) and corynoxeine (**20**) had measured binding affinities below 10 μM at the alpha-2A (K_i_ = 4.8 M) and alpha-2B (K_i_ = 8.4 μM) adrenergic receptors, respectively.

The target prediction evaluation of the kratom alkaloids at the opioid, adrenergic, and serotonin receptors does not aim to or provide a systematic validation of the individual target prediction models within Clarity or SEAware. Rather, the target prediction models are used to guide experimental inquiry into newly identified compounds that lack experimental data. Both Clarity and SEAware contain thousands of targets, and each target would require its own unique validation set of chemicals to assess the predictive performance of each model. While a more rigorous statistical analysis of each target would be advantageous, it is prohibitively time intensive for the context of use and would limit the utility of PHASE as it is intended to provide a rapid *in silico* characterization of an emerging threat.

In summary, PHASE was used to construct *in silico* binding profiles of 25 kratom alkaloids. The binding profiles of the mitragynine congeners (**1–7**) indicate these compounds would likely bind the opioid (mu, kappa, delta), adrenergic (Alpha-2A, 2B, 2C), and serotonin (5-HT1A, 2A) receptors. The binding predictions of mitragynine (**1**) and speciogynine (**2**) were confirmed using radioligand binding assays and quantified the binding affinity at these targets. Similarly, the binding profiles of the pyran-fused mitragynine congeners, ajmalicine (**11**) and tetrahydroalstonine (**12**), were verified and correctly predicted that these compounds would bind serotonin receptors. Not surprisingly, the pyran-fused mitragynine congeners have very strong binding affinity at the adrenergic receptors considering ajmalicine is a drug used to treat high blood pressure[48]. Additionally, the mu opioid molecular docking model correctly predicted the decreased binding affinity of the pyran-fused mitragynine congeners with respect to mitragynine. Docking models for the adrenergic, serotonin receptors, and the κ and δ opioid receptors are being developed. Finally, the binding profiles of the oxindole congeners (**13–25**) correctly indicated some of these compounds bind the opioid receptors but not the adrenergic or serotonin receptors.

## Conclusions

Kratom is a botanical substance that has become popular in the EU and US as a novel, recreational substance for producing a ‘legal high’[5]. Kratom exhibits complex, dose-dependent effects and understanding the pharmacology is confounded by the presence of over 40 alkaloids in the leaves. Evaluating the binding and function of all alkaloids at all potential targets is prohibitively time intensive. Therefore, the rapid generation of *in silico* binding profiles for newly identified compounds provide a guide for prioritizing experimental evaluation.

In this work, *in silico* binding profiles of kratom alkaloids, generated by PHASE, were used to prioritize experimental inquiry into the potential effects of kratom alkaloids beyond the opioid receptors. Specifically, mitragynine and speciogynine, which account for 66 and 7% of kratom’s alkaloid concentration[5], were demonstrated to bind the adrenergic and serotonin receptors. Interestingly, oxidation of mitragynine (**1**) to 7-hydroxymitragynine (**7**) significantly strengthens the binding affinity at the mu opioid receptor but weakens affinity at the adrenergic and serotonin receptors. Other kratom alkaloids, e.g. ajmalicine (**11**) and tetrahydroalstonine (**12**), bind the adrenergic and serotonin receptors but account for much lower concentration than mitragynine. Furthermore, understanding the variability in alkaloid concentration of different kratom products is required for fully evaluating risks associated with kratom. Finally, this work highlights the need for further experimental inquiry of kratom alkaloids beyond the opioid receptors.

## Supporting information

S1 Fig (TIF)

S1 Data (ZIP)
