# Supplementary figures and images for: Evaluating kratom alkaloids using PHASE
Source: PLoS One. 2020 Mar 3;15(3):e0229646. doi: 10.1371/journal.pone.0229646 (PMC7053747; doi:10.1371/journal.pone.0229646)

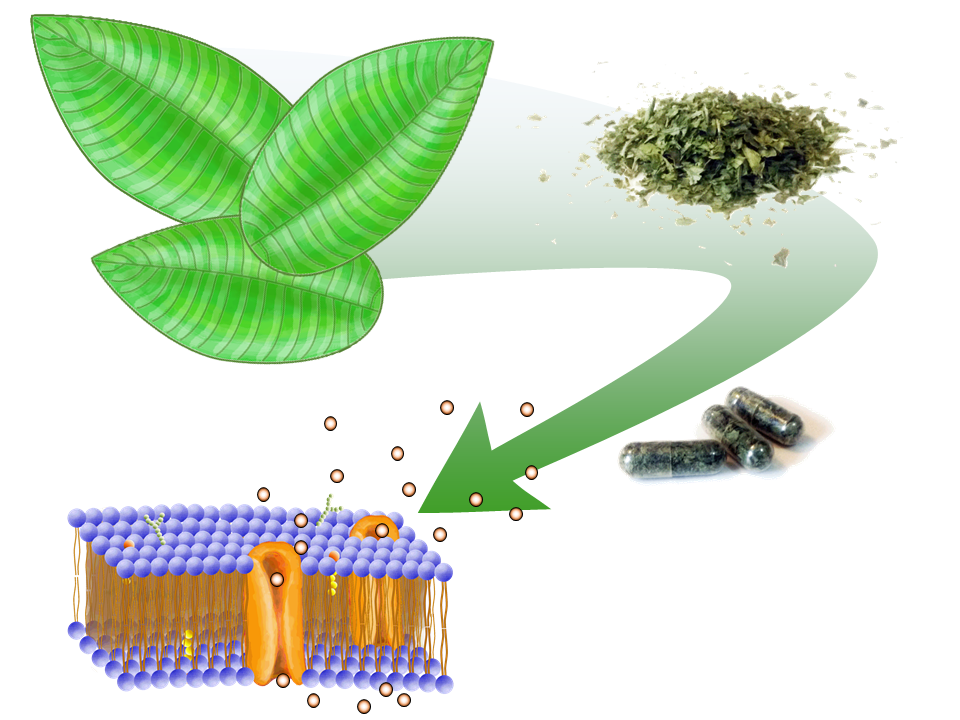

Supplement: S1 Fig — (TIF) [file pone.0229646.s001.tif]
