# Supplementary material for: Evaluating kratom alkaloids using PHASE
Source: PLoS One. 2020 Mar 3;15(3):e0229646. doi: 10.1371/journal.pone.0229646 (PMC7053747; doi:10.1371/journal.pone.0229646)

File ..CHEM\06-18\210618-NCGC00488797-0111-50581.D Tgt Mass (EZX): 398.22

Injection Date : 21-Jun-18, 22:31:49 Seq. Line : 0
Sample Name : NCGC00488797-01 Location : P1-C-09
Acq. Operator : Zina Itkin Inj : 0
Spec. Reported : UV Integration Inj Volume : 1 ul
Acq. Method : C:\Chem32\1\METHODS\FINAL_GRAD_NO_PRINT.M
Analysis Method : C:\Chem32\1\METHODS\FINAL_GRAD_NO_PRINT.M
Sample Info : 0202647462 Easy-Access Method: 'FINAL_GRAD_NO_PRINT' 398.22
Method Info : Long Gradient 4% to 100% ACN over 7 minutes (0.05%TFA)
Luna C18 3.0 x 75 mm

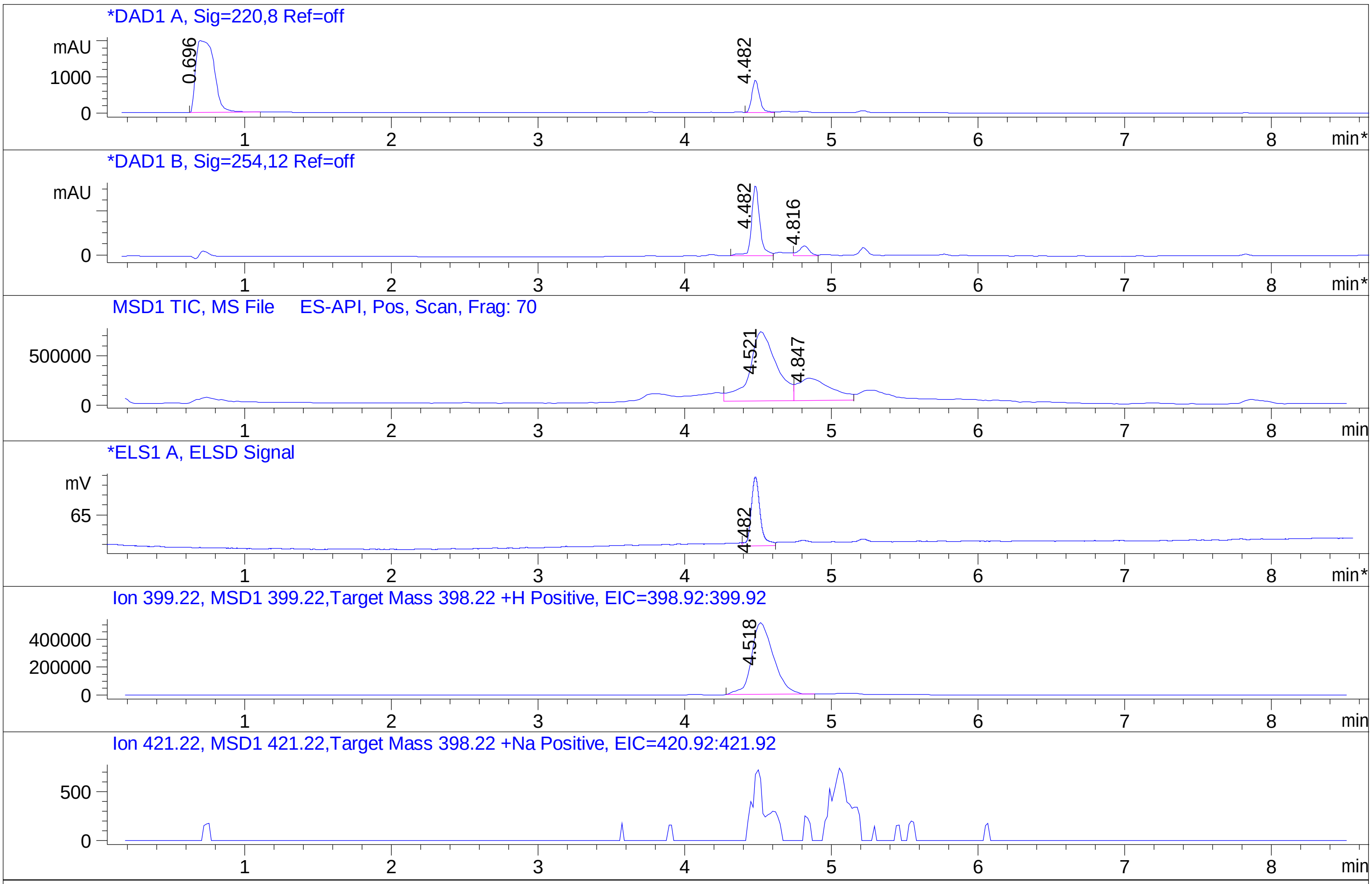

Integration Results for DAD1 A, Sig=220,8 Ref=off

RetTim	Width	Area	Height	Area%	MS(+)
0.70	0.11	17336.03	1998.92	83.87	179
4.48	0.06	3333.05	899.58	16.13	399

Integration Results for DAD1 B, Sig=254,12 Ref=off

RetTim	Width	Area	Height	Area%	MS(+)
4.48	0.06	1255.76	318.72	85.25	399
4.82	0.07	217.29	44.43	14.75	425

Ret. Time: 0.70 <<<< POSITIVE SPECTRA >>>>

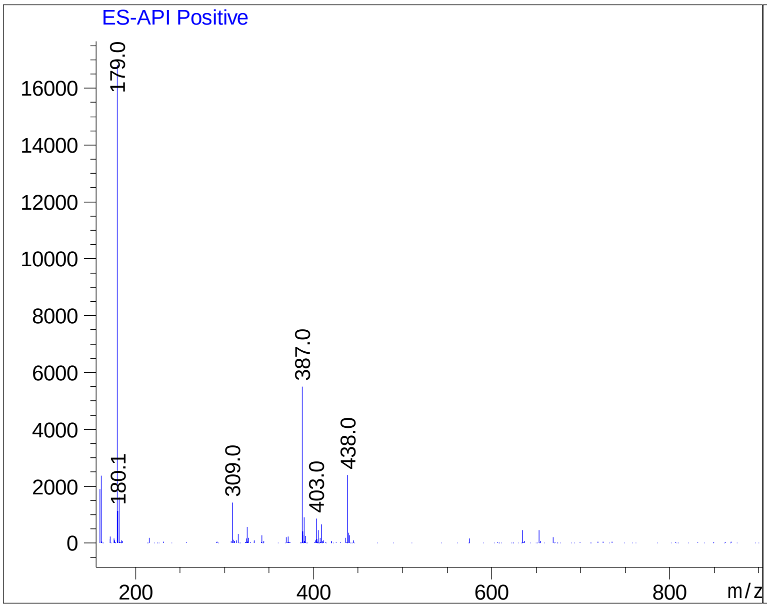

Ret. Time: 4.48

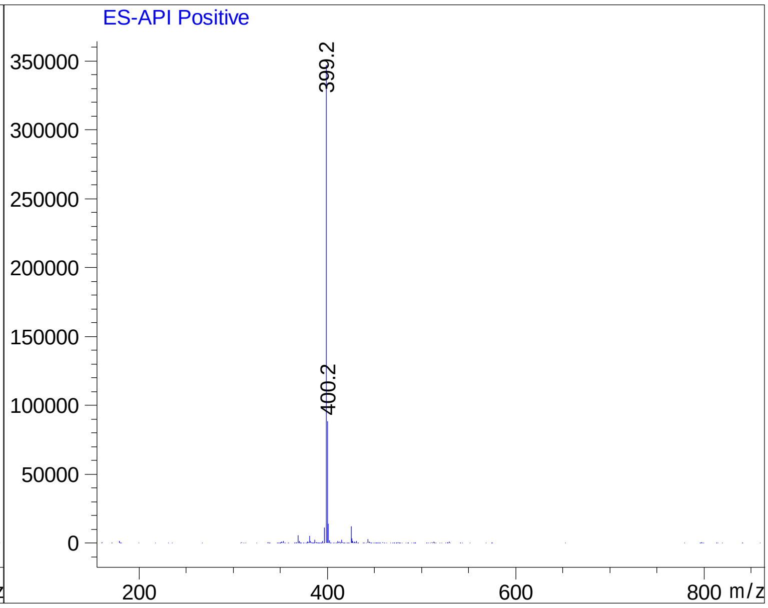

Ret. Time: 4.82

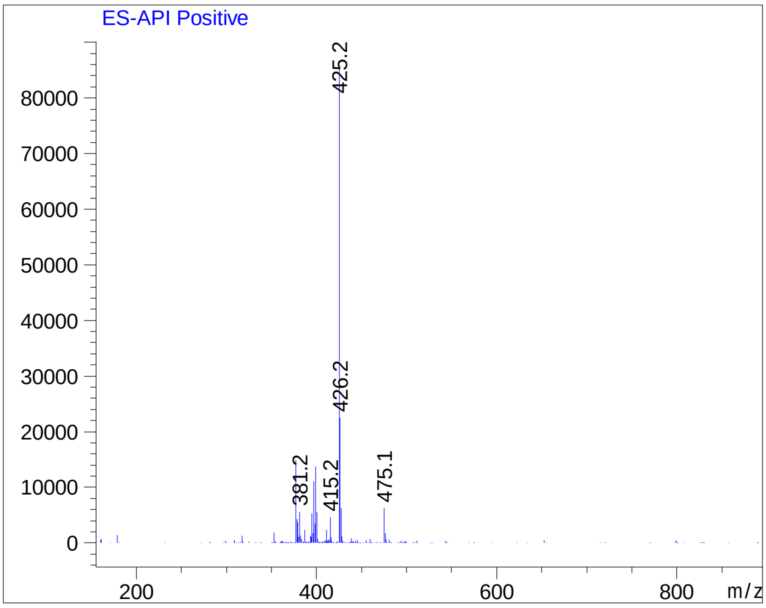

File ..CHEM\06-18\210618-NCGC00488796-0111-50580.D Tgt Mass (EZX): 398.22

Injection Date : 21-Jun-18, 11:41:02   Seq. Line : 0
Sample Name : NCGC00488796-01   Location : P1-C-08
Acq. Operator : Zina Itkin   Inj : 0
Spec. Reported : UV Integration   Inj Volume : 3 ul
Acq. Method : C:\Chem32\1\METHODS\FINAL_GRAD_NO_PRINT.M
Analysis Method : C:\Chem32\1\METHODS\FINAL_GRAD_NO_PRINT.M
Sample Info : 0202647536 Easy-Access Method: 'FINAL_GRAD_NO_PRINT' 398.22
Method Info : Long Gradient 4% to 100% ACN over 7 minutes (0.05%TFA)
Luna C18 3.0 x 75 mm

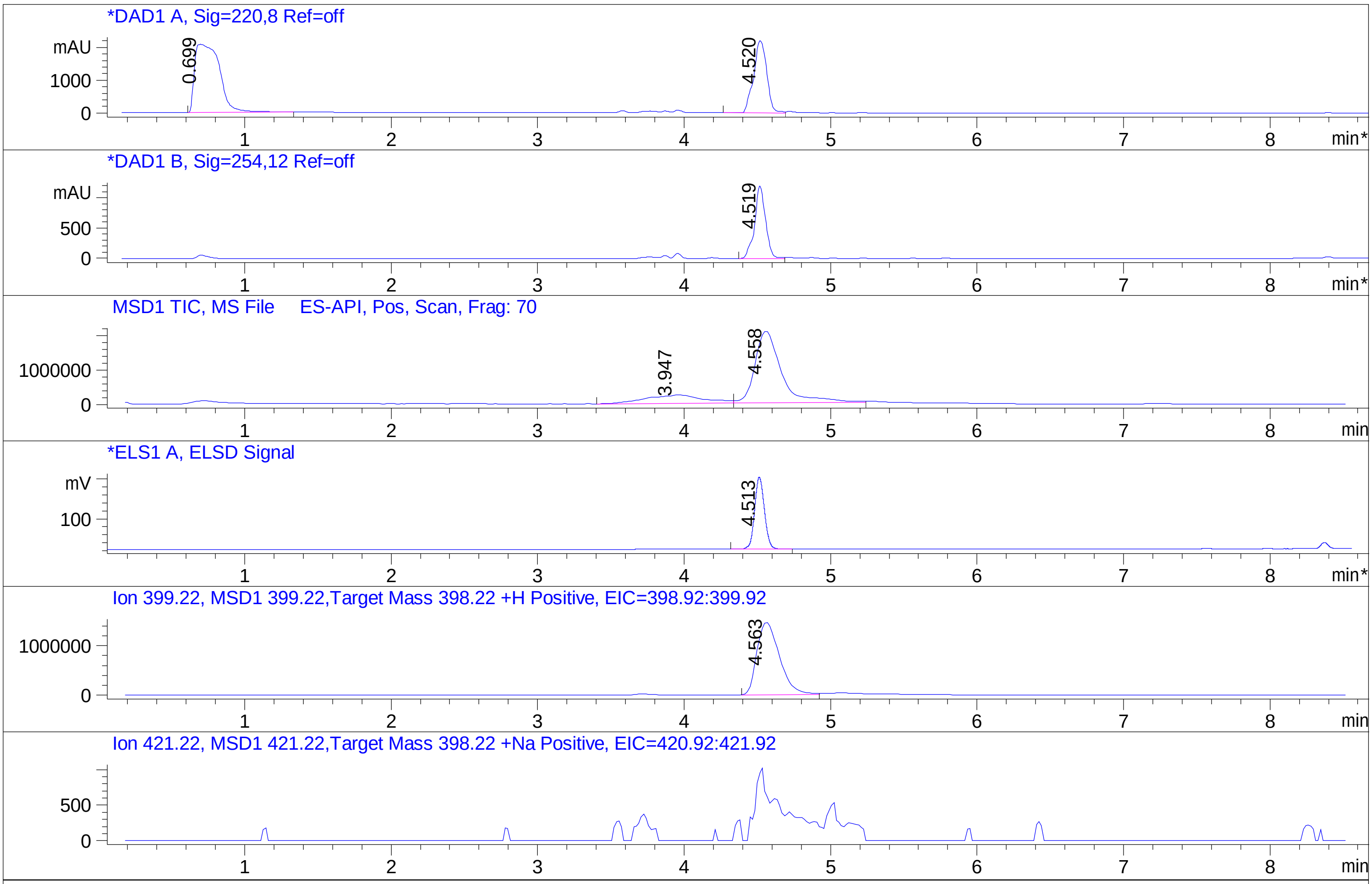

Integration Results for DAD1 A, Sig=220,8 Ref=off

RetTim	Width	Area	Height	Area%	MS(+)
0.70	0.16	24756.13	2080.70	64.20	179
4.52	0.09	13804.11	2207.90	35.80	399

Integration Results for DAD1 B, Sig=254,12 Ref=off

RetTim	Width	Area	Height	Area%	MS(+)
4.52	0.08	6289.36	1204.01	100.00	399

Ret. Time: 0.70 <<<< POSITIVE SPECTRA >>>>   Ret. Time: 4.52

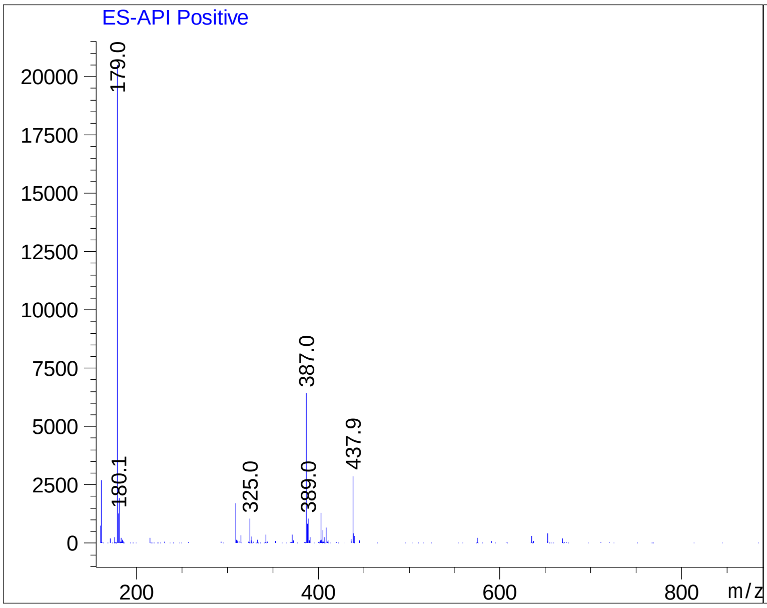

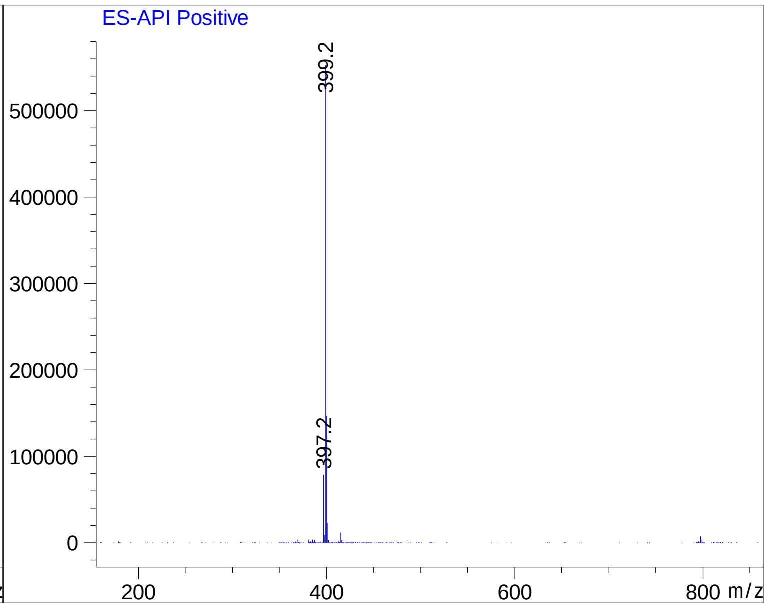

File ..CHEM\06-18\210618-NCGC00488460-0111-50582.D Tgt Mass (EZX): 414.22

Injection Date : 21-Jun-18, 22:41:47   Seq. Line : 0
Sample Name : NCGC00488460-01   Location : P1-D-01
Acq. Operator : Zina Itkin   Inj : 0
Spec. Reported : UV Integration   Inj Volume : 1 ul
Acq. Method : C:\Chem32\1\METHODS\FINAL_GRAD_NO_PRINT.M
Analysis Method : C:\Chem32\1\METHODS\FINAL_GRAD_NO_PRINT.M
Sample Info : 0202647463 Easy-Access Method: 'FINAL_GRAD_NO_PRINT' 414.22
Method Info : Long Gradient 4% to 100% ACN over 7 minutes (0.05%TFA)
Luna C18 3.0 x 75 mm

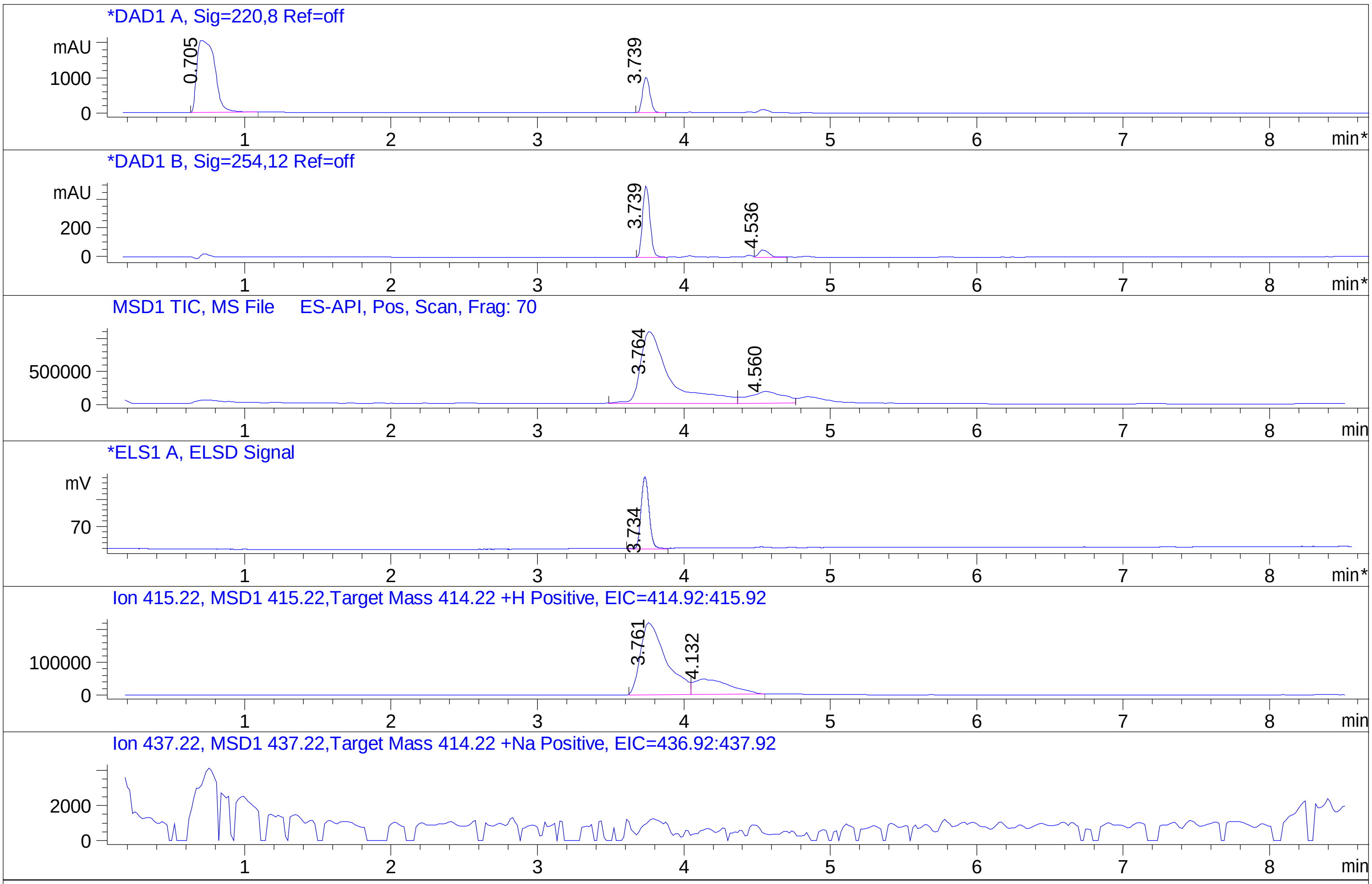

Integration Results for DAD1 A, Sig=220,8 Ref=off

RetTim	Width	Area	Height	Area%	MS(+)
0.70	0.12	17415.40	2046.97	83.11	179
3.74	0.05	3538.08	1009.92	16.89	433

Integration Results for DAD1 B, Sig=254,12 Ref=off

RetTim	Width	Area	Height	Area%	MS(+)
3.74	0.05	1759.83	503.34	87.66	433
4.54	0.08	247.80	50.53	12.34	398

Ret. Time: 0.70 <<<< POSITIVE SPECTRA >>>>

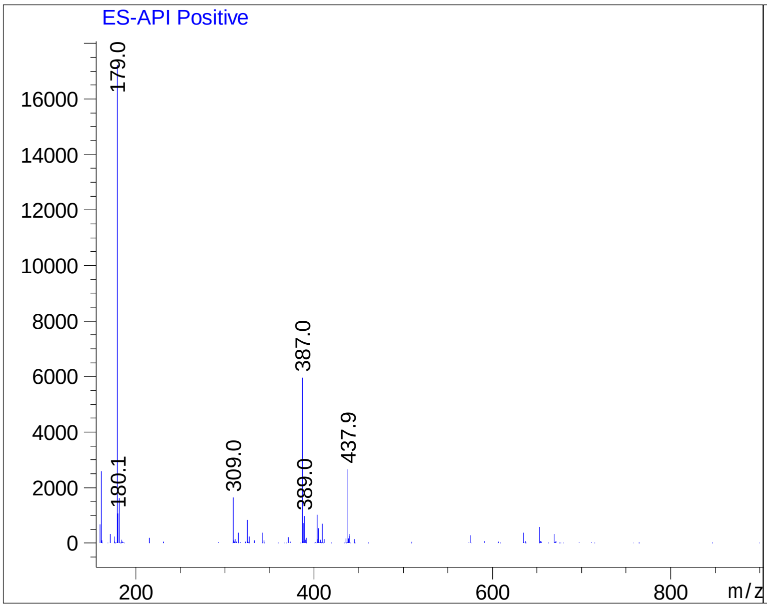

Ret. Time: 3.74

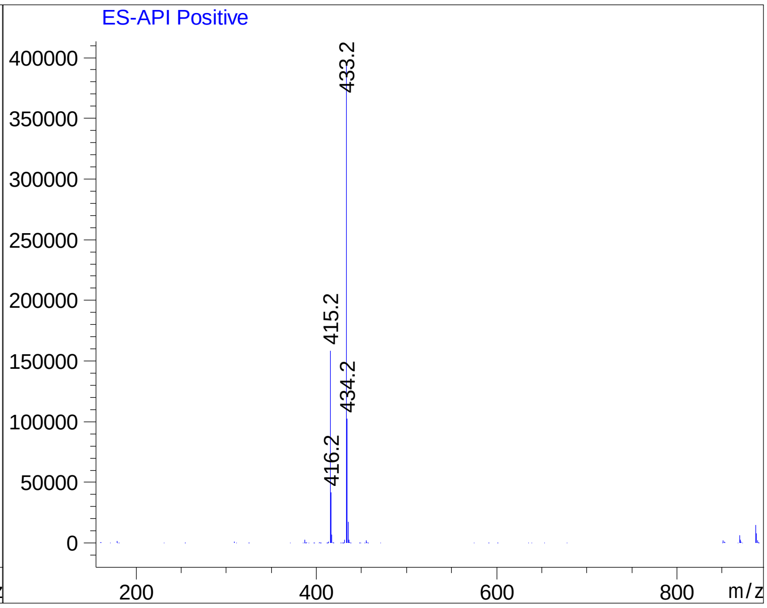

Ret. Time: 4.54

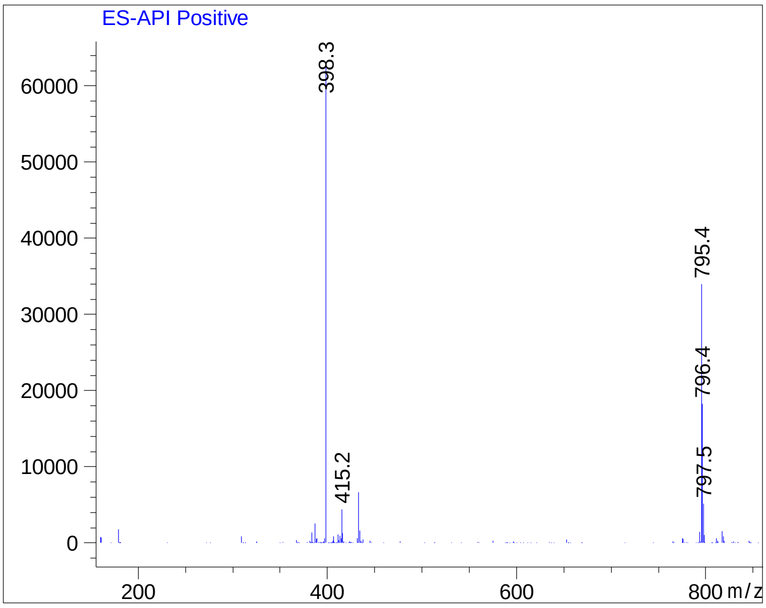

File ..CHEM\06-18\210618-NCGC00163632-0111-50575.D Tgt Mass (EZX): 352.18

Injection Date : 21-Jun-18, 10:50:27 Seq. Line : 0
Sample Name : NCGC00163632-01 Location : P1-C-03
Acq. Operator : Zina Itkin Inj : 0
Spec. Reported : UV Integration Inj Volume : 3 ul
Acq. Method : C:\Chem32\1\METHODS\FINAL_GRAD_NO_PRINT.M
Analysis Method : C:\Chem32\1\METHODS\FINAL_GRAD_NO_PRINT.M
Sample Info : 0059005040 Easy-Access Method: 'FINAL_GRAD_NO_PRINT' 352.18
Method Info : Long Gradient 4% to 100% ACN over 7 minutes (0.05%TFA)
Luna C18 3.0 x 75 mm

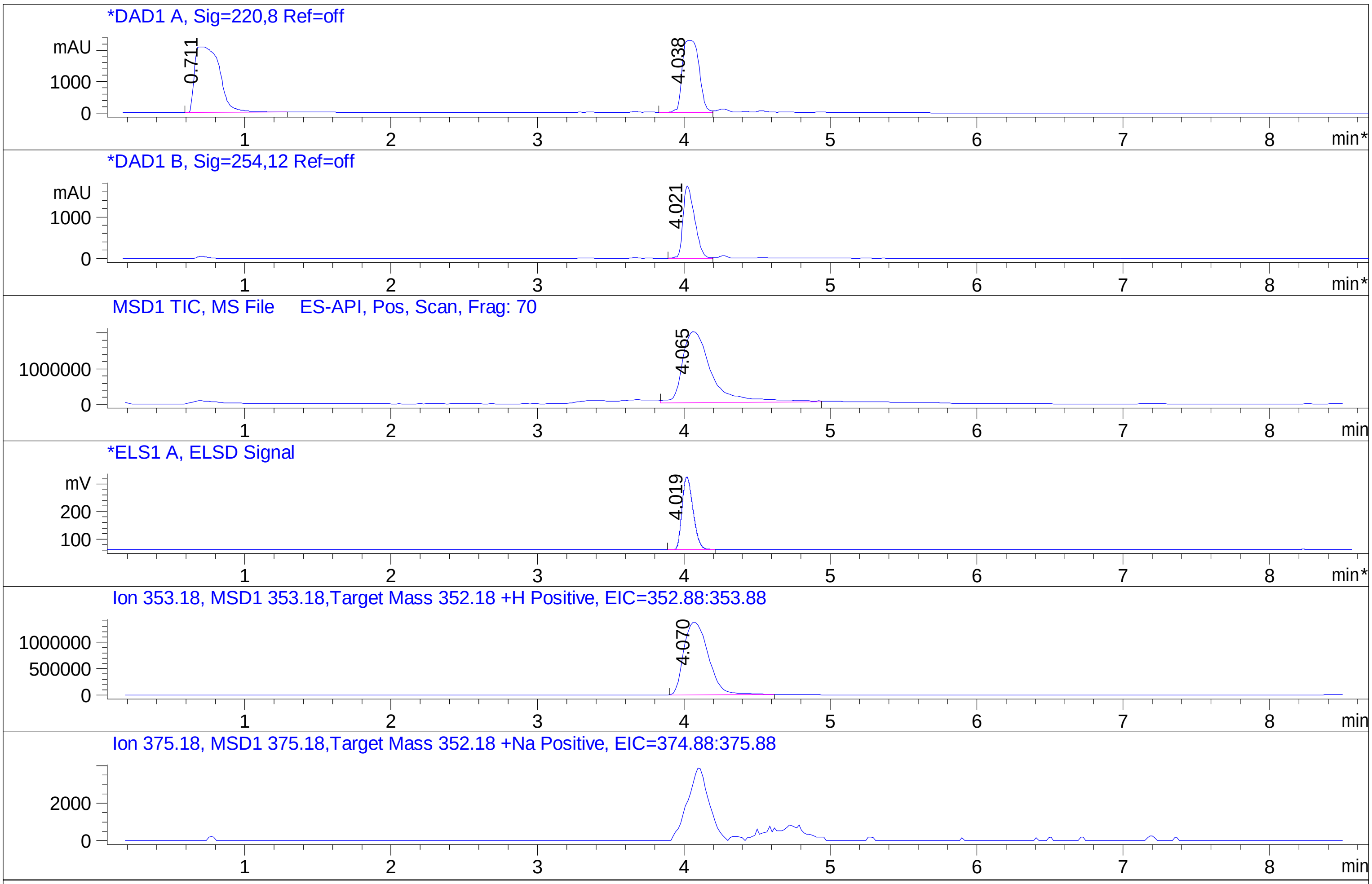

Integration Results for DAD1 A, Sig=220,8 Ref=off

RetTim	Width	Area	Height	Area%	MS(+)
0.71	0.18	24889.07	2094.97	57.30	179
4.04	0.13	18547.92	2302.84	42.70	353

Integration Results for DAD1 B, Sig=254,12 Ref=off

RetTim	Width	Area	Height	Area%	MS(+)
4.02	0.08	9677.41	1753.51	100.00	353

Ret. Time: 0.71 <<<< POSITIVE SPECTRA >>>>

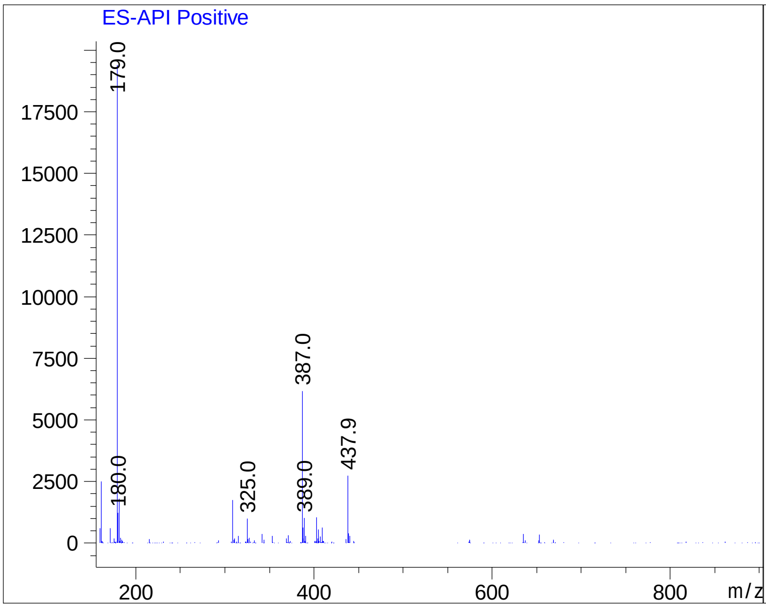

Ret. Time: 4.02

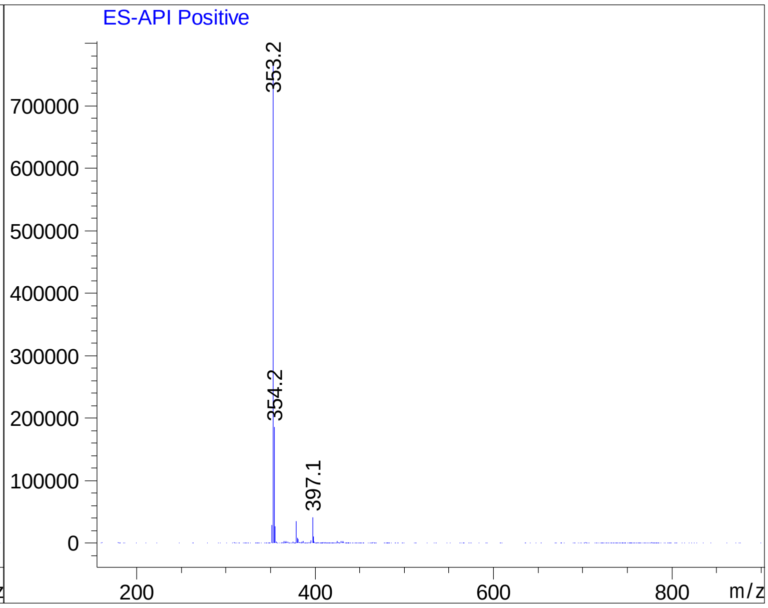

Ret. Time: 4.04

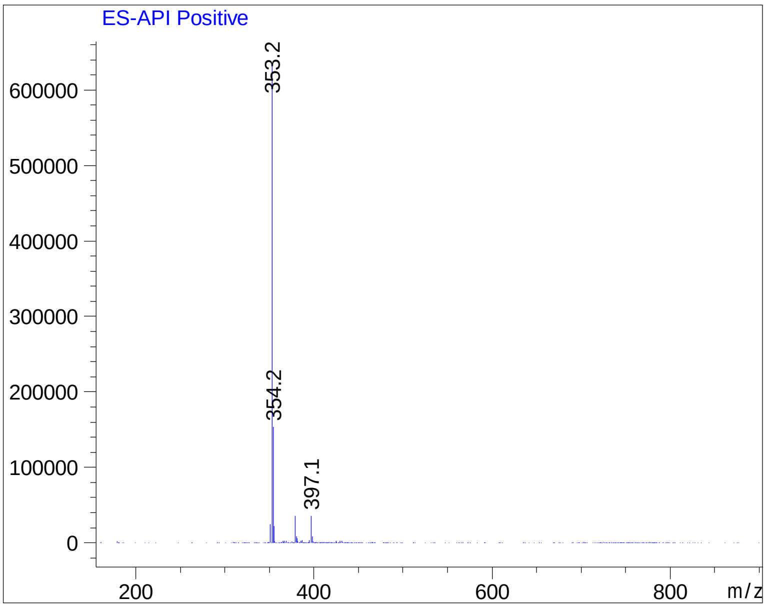

File ..CHEM\06-18\210618-NCGC00163632-0211-50569.D Tgt Mass (EZX): 352.18
Injection Date : 21-Jun-18, 09:50:00 Seq. Line : 0
Sample Name : NCGC00163632-02 Location : P1-B-06
Acq. Operator : Zina Itkin Inj : 0
Spec. Reported : UV Integration Inj Volume : 3 ul
Acq. Method : C:\Chem32\1\METHODS\FINAL_GRAD_NO_PRINT.M
Analysis Method : C:\Chem32\1\METHODS\FINAL_GRAD_NO_PRINT.M
Sample Info : 0202569776 Easy-Access Method: 'FINAL_GRAD_NO_PRINT' 352.18
Method Info : Long Gradient 4% to 100% ACN over 7 minutes (0.05%TFA)
Luna C18 3.0 x 75 mm

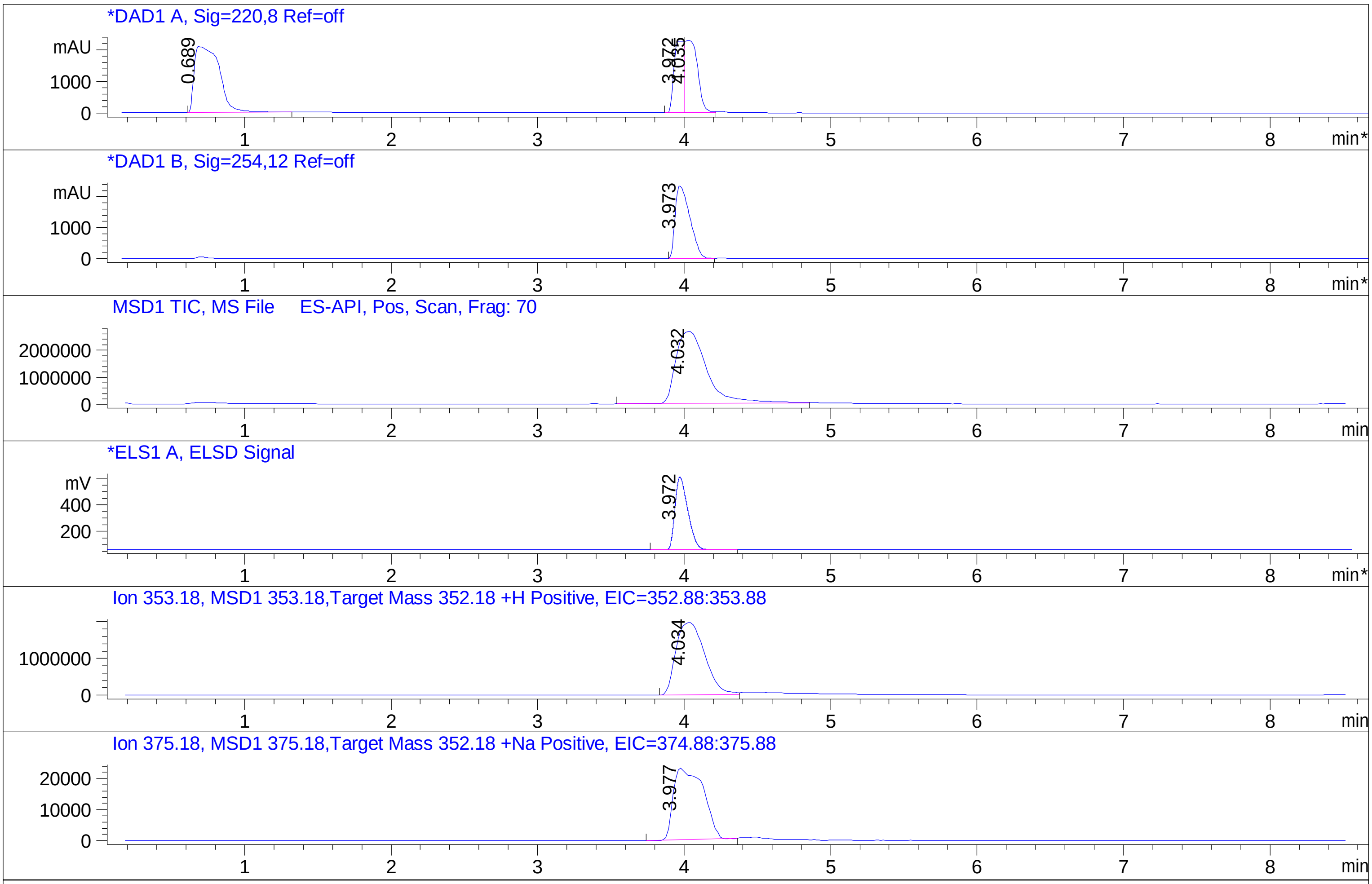

Integration Results for DAD1 A, Sig=220,8 Ref=off

RetTim	Width	Area	Height	Area%	MS(+)
0.69	0.16	24832.56	2079.22	50.56	179
3.97	0.07	9749.72	2264.38	19.85	353
4.04	0.10	14532.65	2280.98	29.59	353

Integration Results for DAD1 B, Sig=254,12 Ref=off

RetTim	Width	Area	Height	Area%	MS(+)
3.97	0.11	16531.07	2356.63	100.00	353

Ret. Time: 0.69 <<<< POSITIVE SPECTRA >>>>

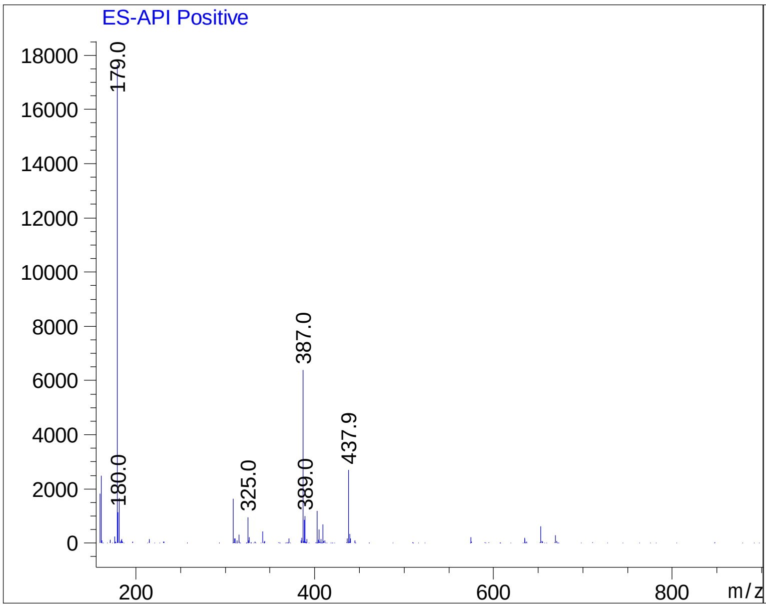

Ret. Time: 3.97

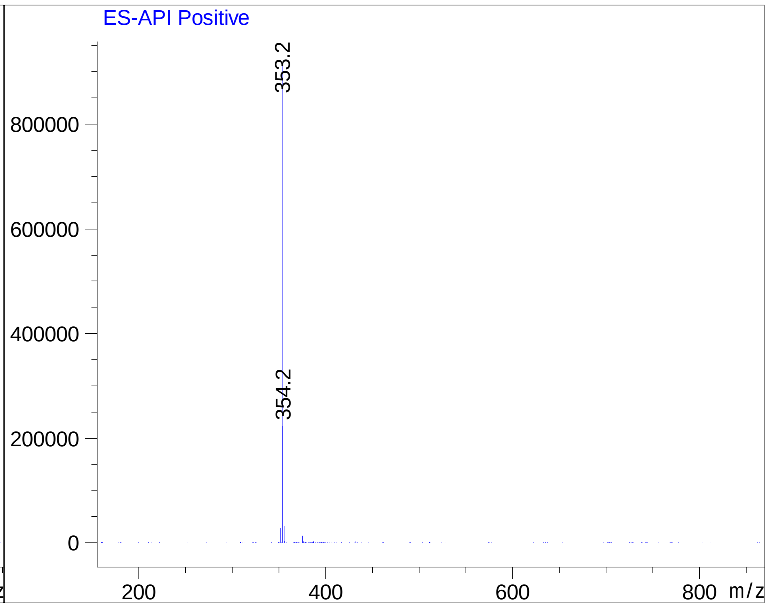

Ret. Time: 4.04

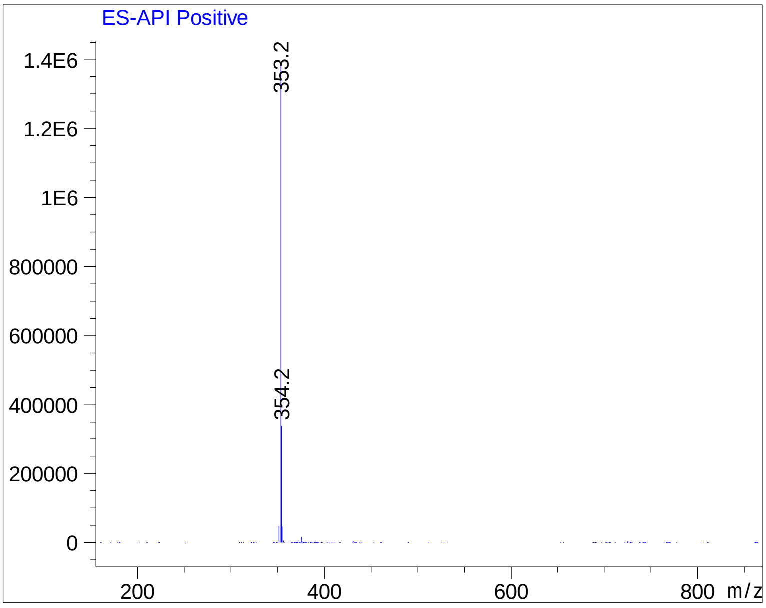

File ..CHEM\06-18\210618-NCGC00482767-0211-50568.D Tgt Mass (EZX): 384.20

Injection Date : 21-Jun-18, 09:40:01   Seq. Line : 0
Sample Name : NCGC00482767-02   Location : P1-B-05
Acq. Operator : Zina Itkin   Inj : 0
Spec. Reported : UV Integration   Inj Volume : 3 ul
Acq. Method : C:\Chem32\1\METHODS\FINAL_GRAD_NO_PRINT.M
Analysis Method : C:\Chem32\1\METHODS\FINAL_GRAD_NO_PRINT.M
Sample Info : 0202656964 Easy-Access Method: 'FINAL_GRAD_NO_PRINT' 384.20
Method Info : Long Gradient 4% to 100% ACN over 7 minutes (0.05%TFA)
Luna C18 3.0 x 75 mm

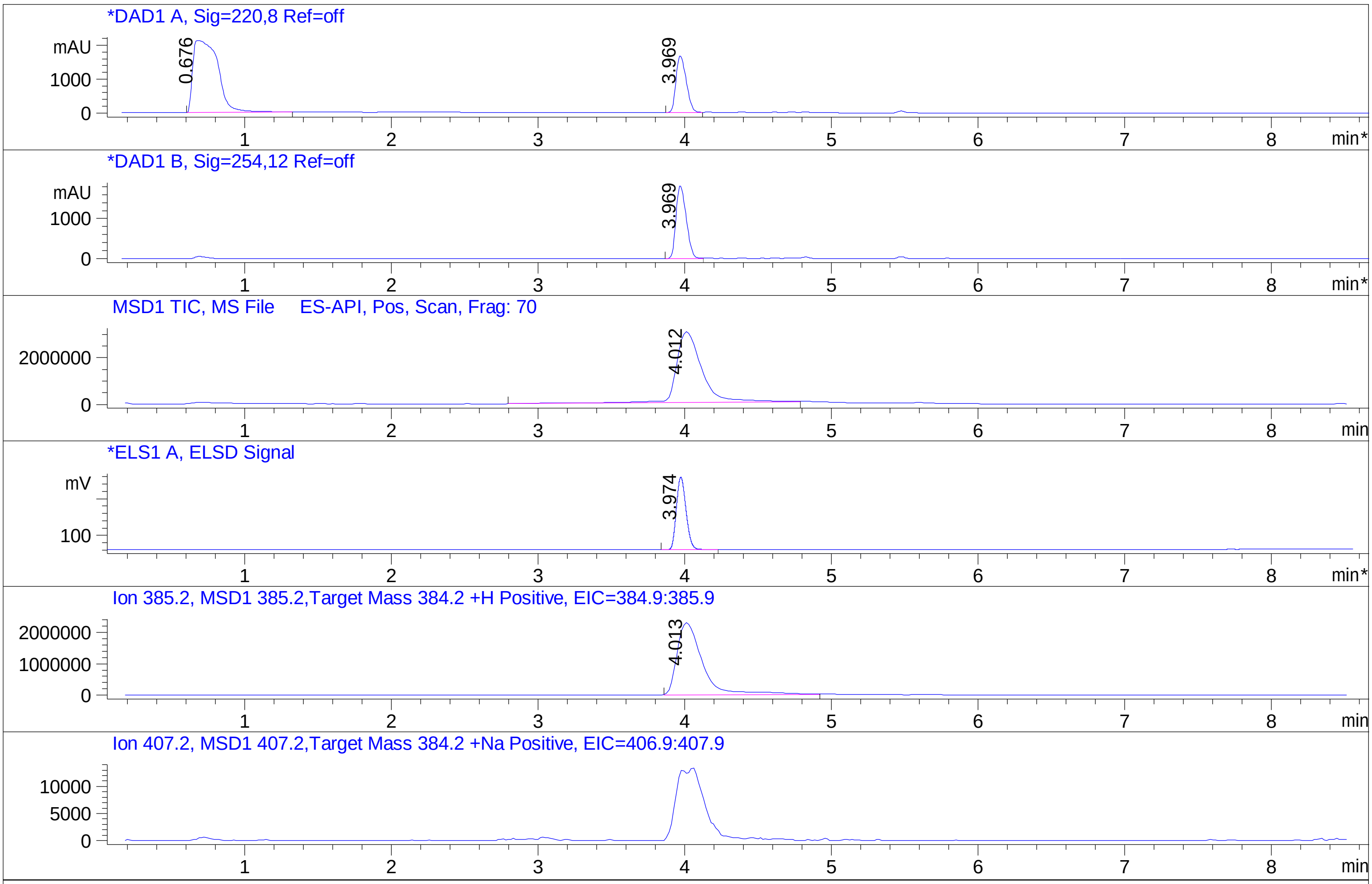

Integration Results for DAD1 A, Sig=220,8 Ref=off

RetTim	Width	Area	Height	Area%	MS(+)
0.68	0.17	25201.36	2127.12	75.46	179
3.97	0.08	8196.20	1677.61	24.54	385

Integration Results for DAD1 B, Sig=254,12 Ref=off

RetTim	Width	Area	Height	Area%	MS(+)
3.97	0.08	8893.80	1832.42	100.00	385

Ret. Time: 0.68 <<<< POSITIVE SPECTRA >>>> Ret. Time: 3.97

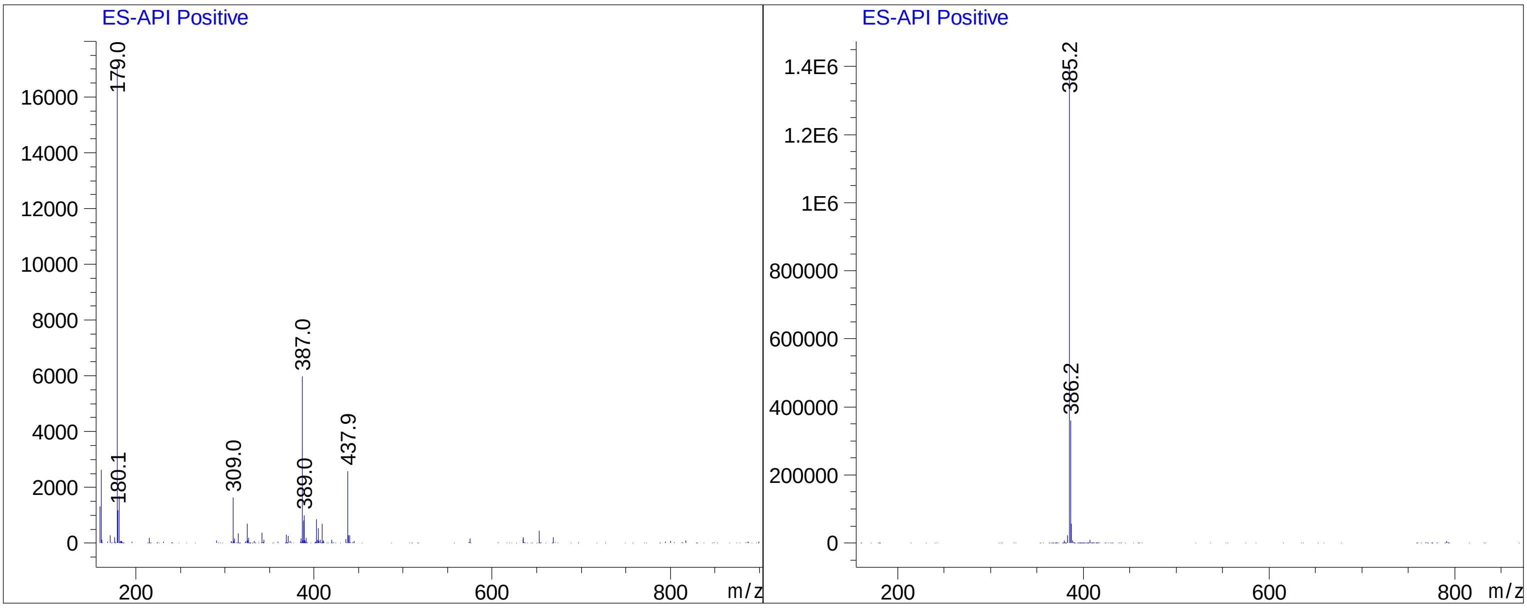

File ..CHEM\06-18\210618-NCGC00482766-0211-50567.D Tgt Mass (EZX): 384.20

Injection Date : 21-Jun-18, 09:30:01   Seq. Line : 0
Sample Name : NCGC00482766-02   Location : P1-B-04
Acq. Operator : Zina Itkin   Inj : 0
Spec. Reported : UV Integration   Inj Volume : 3 ul
Acq. Method : C:\Chem32\1\METHODS\FINAL_GRAD_NO_PRINT.M
Analysis Method : C:\Chem32\1\METHODS\FINAL_GRAD_NO_PRINT.M
Sample Info : 0202656979 Easy-Access Method: 'FINAL_GRAD_NO_PRINT' 384.20
Method Info : Long Gradient 4% to 100% ACN over 7 minutes (0.05%TFA)
Luna C18 3.0 x 75 mm

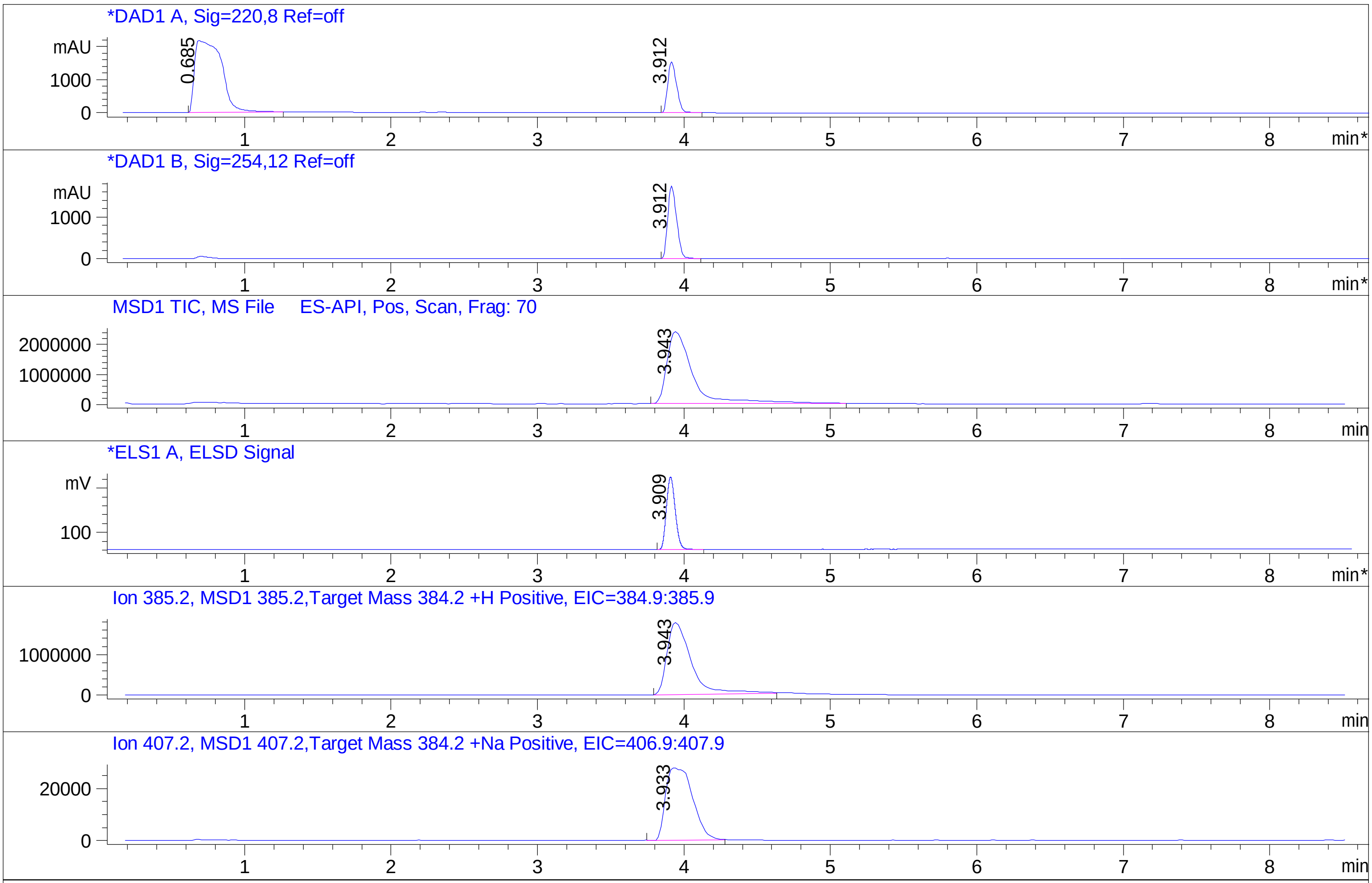

Integration Results for DAD1 A, Sig=220,8 Ref=off

RetTim	Width	Area	Height	Area%	MS(+)
0.68	0.16	27752.10	2179.84	81.01	179
3.91	0.06	6505.98	1553.57	18.99	385

Integration Results for DAD1 B, Sig=254,12 Ref=off

RetTim	Width	Area	Height	Area%	MS(+)
3.91	0.07	7360.88	1746.86	100.00	385

Ret. Time: 0.68 <<<< POSITIVE SPECTRA >>>> Ret. Time: 3.91

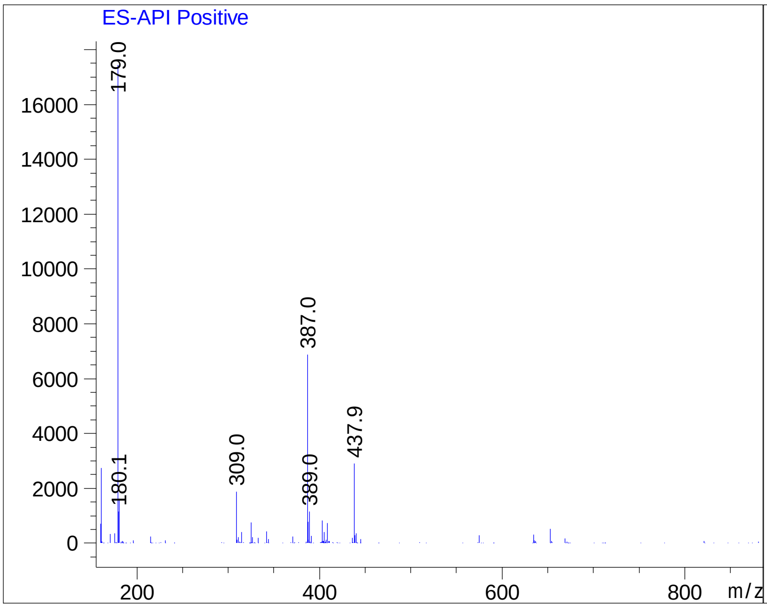

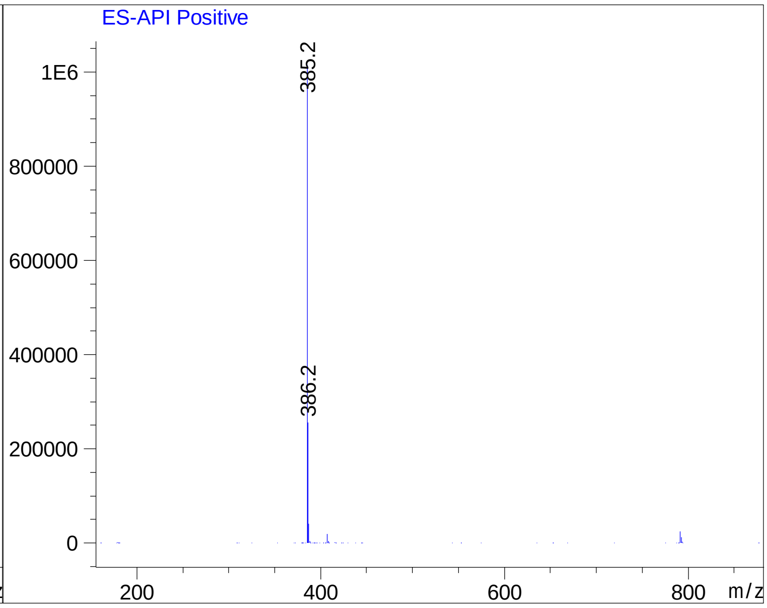

File ..CHEM\06-18\210618-NCGC00482762-0211-50571.D Tgt Mass (EZX): 382.19

Injection Date : 21-Jun-18, 10:10:09 Seq. Line : 0
Sample Name : NCGC00482762-02 Location : P1-B-08
Acq. Operator : Zina Itkin Inj : 0
Spec. Reported : UV Integration Inj Volume : 3 ul
Acq. Method : C:\Chem32\1\METHODS\FINAL_GRAD_NO_PRINT.M
Analysis Method : C:\Chem32\1\METHODS\FINAL_GRAD_NO_PRINT.M
Sample Info : 0194305680 Easy-Access Method: 'FINAL_GRAD_NO_PRINT' 382.19
Method Info : Long Gradient 4% to 100% ACN over 7 minutes (0.05%TFA)
Luna C18 3.0 x 75 mm

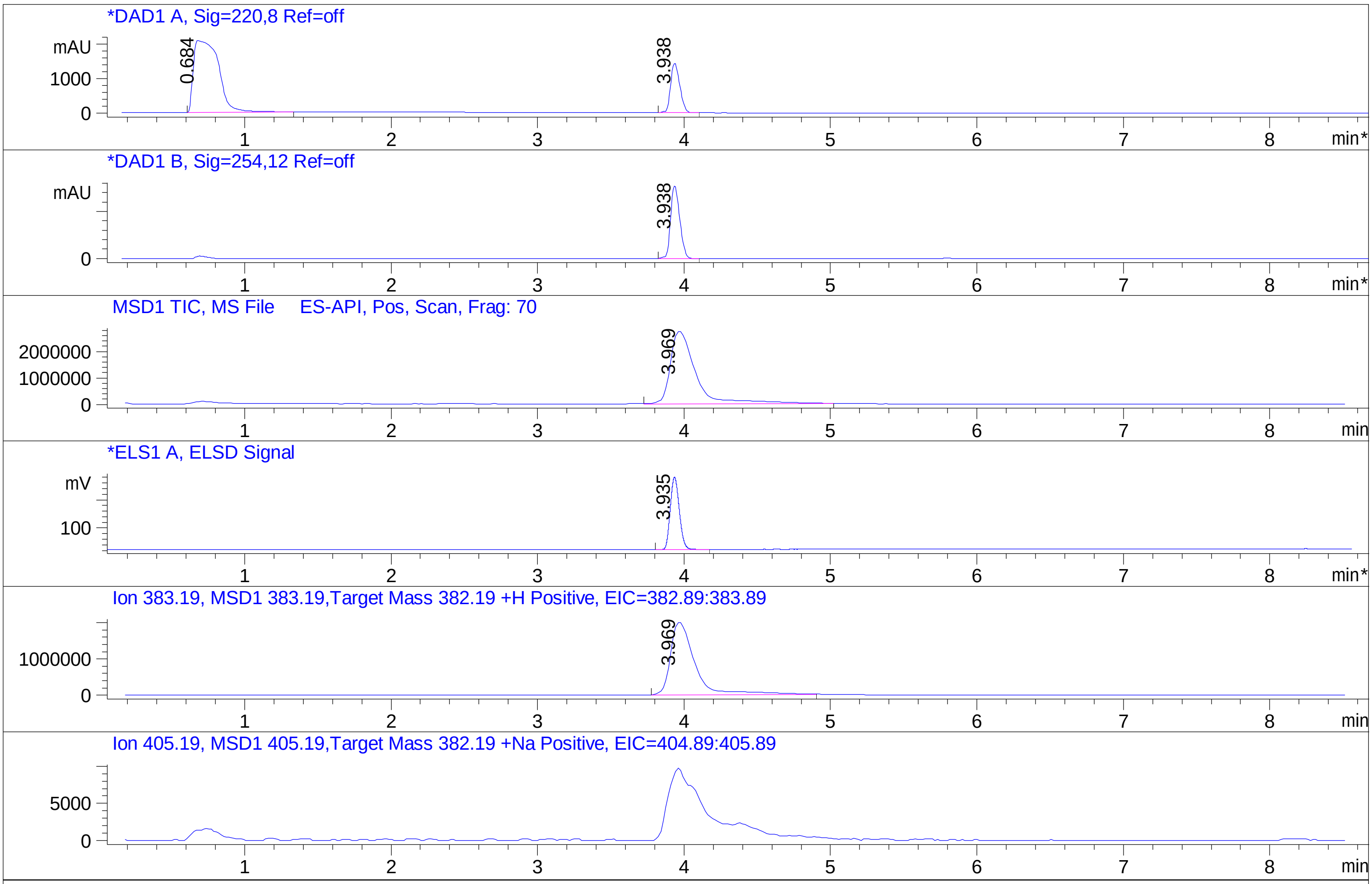

Integration Results for DAD1 A, Sig=220,8 Ref=off

RetTim	Width	Area	Height	Area%	MS(+)
0.68	0.15	24814.32	2084.96	79.82	179
3.94	0.07	6275.15	1436.32	20.18	383

Integration Results for DAD1 B, Sig=254,12 Ref=off

RetTim	Width	Area	Height	Area%	MS(+)
3.94	0.07	6740.53	1553.53	100.00	383

Ret. Time: 0.68 <<<< POSITIVE SPECTRA >>>> Ret. Time: 3.94

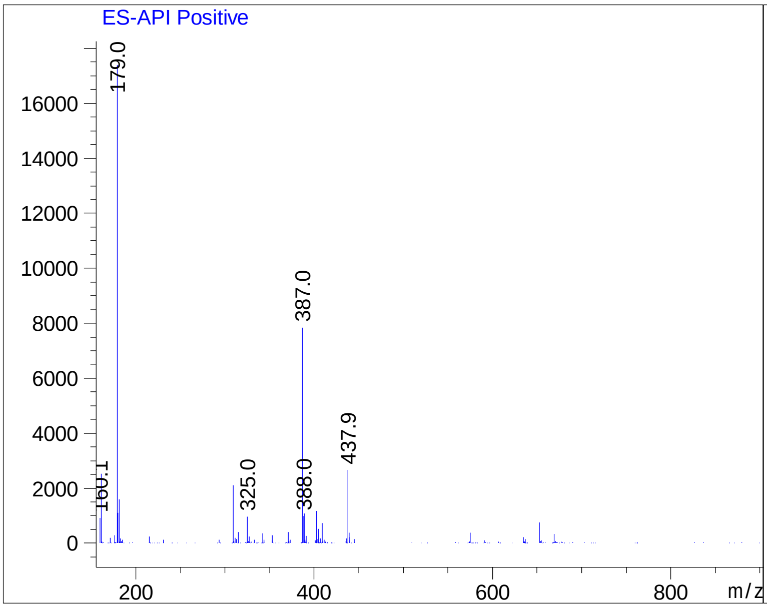

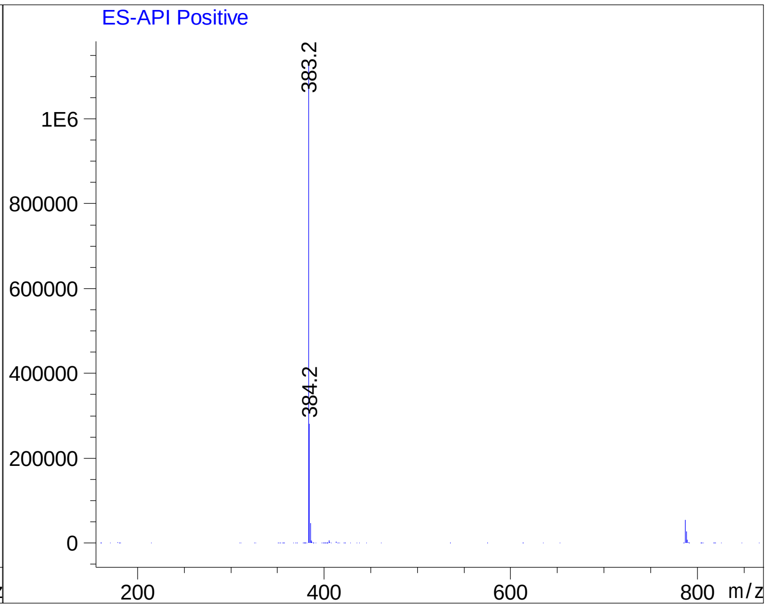

File ..CHEM\06-18\210618-NCGC00482763-0111-50576.D Tgt Mass (EZX): 382.19
Injection Date : 21-Jun-18, 11:00:33 Seq. Line : 0
Sample Name : NCGC00482763-01 Location : P1-C-04
Acq. Operator : Zina Itkin Inj : 0
Spec. Reported : UV Integration Inj Volume : 3 ul
Acq. Method : C:\Chem32\1\METHODS\FINAL_GRAD_NO_PRINT.M
Analysis Method : C:\Chem32\1\METHODS\FINAL_GRAD_NO_PRINT.M
Sample Info : 0202574805 Easy-Access Method: 'FINAL_GRAD_NO_PRINT' 382.19
Method Info : Long Gradient 4% to 100% ACN over 7 minutes (0.05%TFA)
Luna C18 3.0 x 75 mm

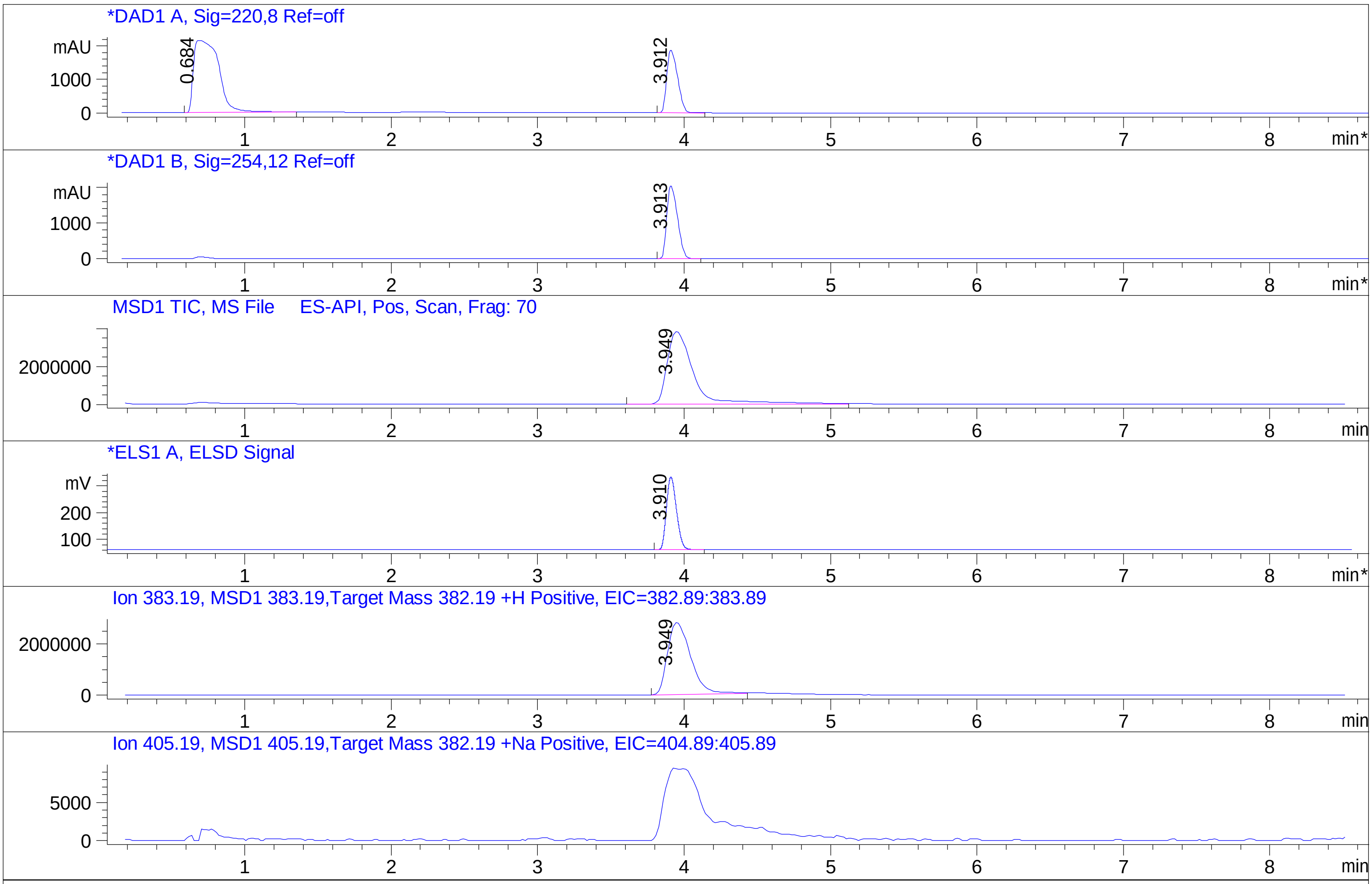

Integration Results for DAD1 A, Sig=220,8 Ref=off

RetTim	Width	Area	Height	Area%	MS(+)
0.68	0.15	25638.18	2150.32	72.59	179
3.91	0.09	9681.22	1874.37	27.41	383

Integration Results for DAD1 B, Sig=254,12 Ref=off

RetTim	Width	Area	Height	Area%	MS(+)
3.91	0.08	10472.01	2051.19	100.00	383

Ret. Time: 0.68 <<<< POSITIVE SPECTRA >>>>

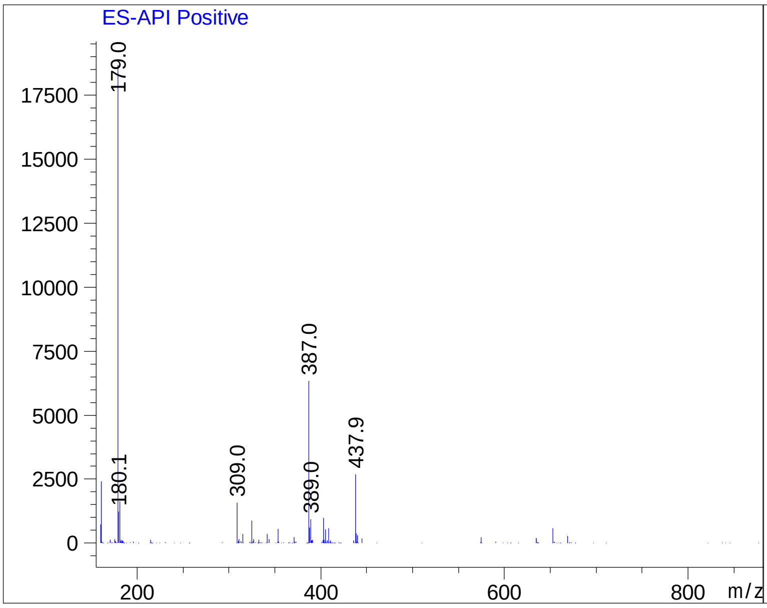

Ret. Time: 3.91

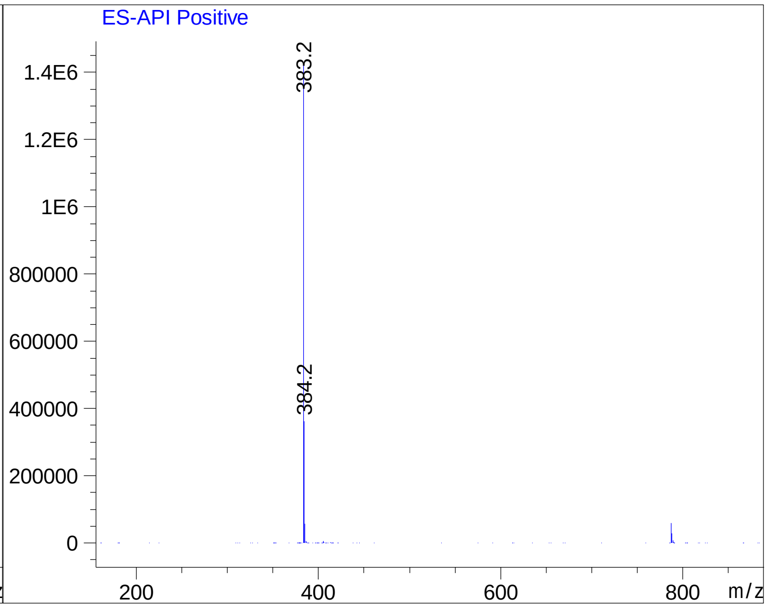

Supplement: S1 Data — (ZIP) [file pone.0229646.s002.zip › SI_compound_qc.pdf]
