# Supplementary material for: Evaluating kratom alkaloids using PHASE
Source: PLoS One. 2020 Mar 3;15(3):e0229646. doi: 10.1371/journal.pone.0229646 (PMC7053747; doi:10.1371/journal.pone.0229646)
Supplement: S1 Data — (ZIP) [file pone.0229646.s002.zip › supporting_information-alkaloid.html]

Supporting Information: Evaluating kratom alkaloids using PHASE


Code 

- Show All Code
- Hide All Code

# Supporting Information: Evaluating kratom alkaloids using PHASE

#### *Christopher R. Ellis, Rebecca Racz, Naomi L. Kruhlak, Marlene T. Kim, Alexey Zakharov, Noel Southall, Edward G. Hawkins, Keith Burkhart, David G. Strauss, Lidiya Stavitskaya*

# Abstract

This document analyzes the data from the similarity analysis, docking simulations, and molecular docking regression model to recreate figures and tables from the main text and provide additional supporting information. The document is generated by knitting ‘SupportingInformation-KratomAnalysis.Rmd’ in Rstudio. It reads data from:

1. fingerprints.txt which contains the MACCS structural keys of the kratom alkaloids and the most similar controlled substances,
2. alkaloid\_best\_poses.txt which contains the docking scores from the 22 kratom alkaloids docked to the mu opioid receptor, and
3. regression\_model.txt which contains the data from the docking simulations of the opioids used to build the regression model for predicting binding affinity (Ellis et al PLoS ONE 2018 13 e0197734).

---

# Structure information

## Mitragynine Congeners

Table S1: Structural information of the mitragynine congeners. The three dimentional structure of each alkaloid was downloaded from Pubchem when available.

| ID | Name | Pubchem ID (CAS) | R1 | R2 | R3 | Stereochemistry |
| --- | --- | --- | --- | --- | --- | --- |
| 1 | Mitragynine | 3034396 | OCH3 | CH2CH3 | H | E, 2S, 3S, 12bS |
| 2 | Speciogynine | 57856495 | OCH3 | CH2CH3 | H | E, 2S, 3R, 12bS |
| 3 | Speciociliatine | 15560576 | OCH3 | CH2CH3 | H | E, 2S, 3S, 12bR |
| 4 | Mitraciliatine | 101281070 | OCH3 | CH2CH3 | H | E, 2S, 3R, 12bR |
| 5 | Paynantheine | 3037629 | OCH3 | CHCH2 | H | E, 2S, 3R, 12bS |
| 6 | Corynantheidine | 3000341 | H | CH2CH3 | H | E, 2S, 3S, 12bS |
| 7 | 7OH-Mitragynine | 44301524 | OCH3 | CH2CH3 | H | E, 2S, 3S, 12bS |
| 8 | Mitragynaline | (132943-52-3) | OCH3 | CH2CH3 | H | 3D structure not used |
| 9 | Mitragynalinic acid | (132943-53-4) | OCH3 | CH2CH3 | CHO | 3D structure not used |
| 10 | Corynantheidalinic acid | - | H | CH2CH3 | CHO | 3D structure not used |
| 11 | Ajmalicine | 441975 | - | - | - | 1S, 15R, 16S,20S |
| 12 | Tetrahydroalstonine | 72340 | - | - | - | 1S, 15S, 16S,20S |

## Pseudoindoxyl Congeners

Table S2: Structural information of the pseudoindoxyl congeners. The three dimentional structure of each alkaloid was downloaded from Pubchem when available.

| ID | Name | Pubchem ID (CAS) | R1 | R2 | Stereochemistry |
| --- | --- | --- | --- | --- | --- |
| 13 | Corynoxine | 10475115 | H | CH2CH3 | E, 3S, 6’S, 7’S, 8’aS |
| 14 | Corynoxine B | 10091424 | H | CH2CH3 | E, 3R, 6’S, 7’S, 8’aS |
| 15 | Rhynchophylline | 5281408 | H | CH2CH3 | E, 3R, 6’R, 7’S, 8’aS |
| 16 | Isorhynchophylline | 3037048 | H | CH2CH3 | E, 3S, 6’R, 7’S, 8’aS |
| 17 | Speciofoline | 5379742 | OH | CH2CH3 | E, 3R, 6’S, 7’R, 8’aR |
| 18 | Isospeciofoline | 101289836 | OH | CH2CH3 | E, 3S, 6’S, 7’S, 8’aR |
| 19 | Mitrafoline | 5379743 | OH | CH2CH3 | Z, 3S, 6’S, 7’R, 8’aR |
| 20 | Corynoxeine | 44568160 | H | CHCH2 | E, 3R, 6’R, 7’S, 8’aS |
| 21 | Isocorynoxeine | 3037448 | H | CHCH2 | E, 3S, 6’R, 7’S, 8’aS |
| 22 | Mitraphylline | 94160 | - | - | 1S, 4aS, 5aS, 6R, 10aR |
| 23 | Isomitraphylline | 11726520 | - | - | 1S, 4aS, 5aS, 6S, 10aR |
| 24 | Speciophylline | 168985 | - | - | 1S, 4aS, 5aR, 6S, 10aS |
| 25 | Isospeciophylline | - | - | - | 1S, 4aS, 5aR, 6R, 10aS |

# Structural similarity analysis

```
#This section reads in 'fingerprints.txt' and recreates Table 1 from the main text.
# read fingerprints
fingerprints <- read.delim('fingerprints.txt', sep ='\t', header = TRUE)
fingerprints$keys <- as.character(fingerprints$keys)
#fingerprints

# convert the MACCS keys to a list of intergers
# alkaloid keys
 c1 <- as.numeric(unlist(strsplit( fingerprints$keys[1], split = ' '))) #mitragynine
 c5 <- as.numeric(unlist(strsplit( fingerprints$keys[2], split = ' '))) #paynantheine
 c6 <- as.numeric(unlist(strsplit( fingerprints$keys[3], split = ' '))) #corynanthedine
 c7 <- as.numeric(unlist(strsplit( fingerprints$keys[4], split = ' '))) #hydroxymitragynine
 c8 <- as.numeric(unlist(strsplit( fingerprints$keys[5], split = ' '))) #mitragynaline
 c9 <- as.numeric(unlist(strsplit( fingerprints$keys[6], split = ' '))) #mitragynalinic acid

c10 <- as.numeric(unlist(strsplit( fingerprints$keys[7], split = ' '))) #corynantheidalinic acid
c11 <- as.numeric(unlist(strsplit( fingerprints$keys[8], split = ' '))) #ajmalicine
c13 <- as.numeric(unlist(strsplit( fingerprints$keys[9], split = ' '))) #corynoxine
c17 <- as.numeric(unlist(strsplit(fingerprints$keys[10], split = ' '))) #speciofoline
c20 <- as.numeric(unlist(strsplit(fingerprints$keys[11], split = ' '))) #corynoxeine
c22 <- as.numeric(unlist(strsplit(fingerprints$keys[12], split = ' '))) #mitraphylline
  
# controlled substances
  acetorphine_keys <- as.numeric(unlist(strsplit(fingerprints$keys[13], split = ' ')))
  nicocodeine_keys <- as.numeric(unlist(strsplit(fingerprints$keys[14], split = ' ')))
 remifentanil_keys <- as.numeric(unlist(strsplit(fingerprints$keys[15], split = ' ')))
 nicomorphine_keys <- as.numeric(unlist(strsplit(fingerprints$keys[16], split = ' ')))
     thebacon_keys <- as.numeric(unlist(strsplit(fingerprints$keys[17], split = ' ')))
lysergic_acid_keys <- as.numeric(unlist(strsplit(fingerprints$keys[18], split = ' ')))
```

Main text Table 1: Structural similarity analysis of the alkaloids with respect to mitragynine and the controlled substance database. The MACCS166 molecular fingerprint only considers two-dimensional structure. Therefore, the twenty-five alkaloids under consideration map to 12 unique chemical structures.

| ID | Name | Number of MACCS166 | Similarity w.r.t. mitragynine, Tc | Most similar controlled substance | Similarity w.r.t Controlled substance |
| --- | --- | --- | --- | --- | --- |
| 1-4 | Mitragynine, Speciogynine, Speciociliatine, Mitraciliatine | 63 | 1(63) | acetorphine | 0.7432432 (55) |
| 5 | Paynantheine | 60 | 0.921875(59) | nicocodeine | 0.7101449 (49) |
| 6 | Corynantheidine | 58 | 0.9206349(58) | acetorphine | 0.6986301 (51) |
| 7 | 7-hydroxy mitragynine | 71 | 0.8356164(61) | acetorphine | 0.7792208 (60) |
| 8 | Mitragynaline | 60 | 0.8636364(57) | nicocodeine | 0.6619718 (47) |
| 9 | Mitragynalinic Acid | 58 | 0.890625(57) | nicocodeine | 0.7058824 (48) |
| 10 | Corynantheidalinic acid | 55 | 0.84375(54) | lysergic acid | 0.7118644 (42) |
| 11-12 | Ajamlicine, Tetrahydroalstonine | 57 | 0.875(56) | nicocodeine | 0.7164179 (48) |
| 13-16 | Corynoxine, Corynoxine B, Rhynchophylline, Isorhynchophylline | 67 | 0.8055556(58) | remifentanil | 0.7222222 (52) |
| 17-19 | Speciofoline, Isospeciofoline, Mitrafoline | 71 | 0.8108108(60) | acetorphine | 0.7564103 (59) |
| 20-21 | Corynoxeine, Isocorynoxeine | 63 | 0.7260274(53) | nicomorphine | 0.6575342 (48) |
| 22-25 | Mitraphylline, Isomitraphylline, Speciophylline, Isospeciophylline | 65 | 0.7534247(55) | thebacon | 0.7222222 (52) |

# Molecular docking results

```
#This section reads in 'alkaloid_best_poses.txt' and recreates Figure 3A from the main text.
#Read in data from the alkaloid docking simulations
docking_results <- read.table('alkaloid_best_poses.txt', header = TRUE)

#Calculate the mean and standard deviation of the best poses for each compound
alkaloid_means <- tapply(docking_results$S , docking_results$name, mean)
alkaloid_sd    <- tapply(docking_results$S , docking_results$name, sd)
alkaloid_ID    <- tapply(docking_results$ID, docking_results$name, mean)

alkaloid_means  <- as.data.frame(alkaloid_means)
alkaloid_sd     <- as.data.frame(alkaloid_sd)
alkaloid_ID     <- as.data.frame(alkaloid_ID)

df_alkaloids    <- cbind(alkaloid_ID, alkaloid_means, alkaloid_sd)

#Sorts df_alkaloids by ascending order of ID
df_alkaloids <- df_alkaloids[order(df_alkaloids$alkaloid_ID),]

# Normalize docking score with respect to mitragynine
df_alkaloids$score_wrt_mitragynine <- df_alkaloids$alkaloid_means - df_alkaloids$alkaloid_means[1]
#df_alkaloids

plot(df_alkaloids$alkaloid_ID, df_alkaloids$score_wrt_mitragynine, ylab='delta G', xlab='Compound Number', 
     pch=18, cex=2, ylim = c(-0.2,2.2))

abline( v=10.5, col='lightpink')
abline( v=12.5, col='lightblue')
abline( v=21.5, col='gray')
```

S1 Fig (Main text Figure 2): Molecular docking evaluation of the alkaloids at the mu opioid receptor. The docking score of the mitragynine congeners (compounds 1-7), pyran-fused mitragynine congeners (compounds 11-12), pseuedoindoxyl congeners (compounds 13-21), and the pyran-fused pseuedoindoxyl congeners (compounds 22-25) normalized to mitragynine.

# Regression model

```
#This section reads in 'regression_model.txt' and recreates Figure 4 from the main text.
#Read in Data for the regression model
raw_model_data <- read.table('regression_model.txt', header=TRUE)

model_score  <- tapply(raw_model_data$S,   raw_model_data$name, mean)
model_Ki     <- tapply(raw_model_data$Ki,  raw_model_data$name, mean)
model_ID     <- tapply(raw_model_data$ID,  raw_model_data$name, mean)
model_score <- as.data.frame(model_score)
model_Ki    <- as.data.frame(model_Ki)
model_ID    <- as.data.frame(model_ID)

#Calculate logKi
model_logKi <- log10(model_Ki)

#Build table
model_info <- cbind(model_ID, model_Ki, model_logKi, model_score)
colnames(model_info)   <- c('model_ID'    , 'model_Ki'   , 'model_logKi'       ,'model_score')
model_info <- model_info[order(model_info$model_ID),]

# Build linear regression model
model.lm <- lm(model_logKi ~ model_score, data = model_info)
#summary(model.lm)
#summary(model.lm)$r.squared

# Initialize array to make confidence and predicivity intervals 
new.dat <- data.frame(model_score=seq(-12, -6, 0.1))

# Calculates confidence and predictivity intervals
#con90  <- as.data.frame(predict(model.lm, newdata = new.dat, interval = 'confidence', level=0.90))
pred70 <- as.data.frame(predict(model.lm, newdata = new.dat, interval = 'prediction', level=0.7))

model_intercept <- as.numeric(model.lm$coefficients[1])
model_slope     <- as.numeric(model.lm$coefficients[2])

#Make plot of regression model
plot(model_info$model_score, model_info$model_logKi, col='black', pch=19, cex=1.,
     xlim=c(-12.0,-6.0),ylab='log(Ki) [nM]', xlab='Score', main='Mu receptor', ylim = c(-2, 6))
axis(side =2, at = c(-1,1,3,5))
abline(lm(model_info$model_logKi~model_info$model_score))

points(new.dat$model_score, pred70$lwr, type = 'l', col ='gray', lwd=3)
points(new.dat$model_score, pred70$upr, type = 'l', col ='gray', lwd=3)
points(model_info$model_score, model_info$model_logKi, col='black', pch=19, cex=1.)
```

S2: Binding affinity regression model.

# Binding Affinity Predictions

```
#The measured Ki of mitragynine at the mu opioid receptor measured by PDSP is 740 nM
#The shift calculation sets mitragynine ADS to be correct

#PDSP measurements under 10,000 nM
PDSP_mitragynine_Ki        <-   740
PDSP_7oh_mitragyinine_Ki   <-    70
PDSP_paynantheine_Ki       <-   780
PDSP_speciogynine_Ki       <-  1000 
PDSP_ajmalicine_Ki         <-  8960
PDSP_corynoxine_b_Ki       <-  1550
PDSP_isorhynchophylline_Ki <-   540 

#PDSP greater than 10,000 nM
PDSP_isocorynoxeine_Ki     <- 10000
PDSP_corynoxeine_Ki        <- 10000
PDSP_tetrahydroalstonine_Ki<- 10000

# center mitragynine
calculated_correct_mitragyine_score <- (log10(PDSP_mitragynine_Ki) - model_intercept)/model_slope
#calculated_correct_mitragyine_score

adjusted_score <- df_alkaloids$alkaloid_means[1] - calculated_correct_mitragyine_score

df_alkaloids$normalized_score <- df_alkaloids$alkaloid_means - adjusted_score

# Calculate the predicted scores
df_alkaloids$pred_logki <- model_slope*df_alkaloids$normalized_score + model_intercept
df_alkaloids$pred_ki    <- 10**(df_alkaloids$pred_logki)
```

```
#Panel A of the Binding Results
   fentanyl_score  <- data.frame(model_score=model_info$model_score[4])
carfentanil_score  <- data.frame(model_score=model_info$model_score[6])

   fentanyl_score_conf <- predict(model.lm, newdata =    fentanyl_score,  interval = 'prediction', level = 0.7)
carfentanil_score_conf <- predict(model.lm, newdata = carfentanil_score,  interval = 'prediction', level = 0.7)

#calculate confidence intervals for kratom alkaloids using regression model
confidence_pred <- as.data.frame(predict(model.lm, newdata =  data.frame((model_score=df_alkaloids$normalized_score)),  
                                         interval = 'prediction', level = 0.7))

df_alkaloids <- cbind(df_alkaloids, confidence_pred)
#df_alkaloids

par(mar=c(5,8,4,2))
plot(log10(PDSP_7oh_mitragyinine_Ki), 15, ylim=c(-2.5, 12.5), xlim=c(-2,7), ylab='', 
     xlab='log(Ki) [nM]', cex=2, pch=18, col ='white', yaxt='n')

#graph_stuff
fent_cex <- 1.5
fent_pch <- 15

PDSP_cex  <- 2
PDSP_pch1 <- 18
conf_cex  <- 1.

#-----------------------------------------------------

#carfentanil
car_pos = 12
points(model_info$model_logKi[6], car_pos, cex=fent_cex, pch=fent_pch, col ='black')

#fentanyl
fent_pos = 11
points(model_info$model_logKi[3], fent_pos, cex=fent_cex, pch=fent_pch, col ='black')

#-----------------------------------------------------

#mitragynine
mitragynine_pos = 10
points(log10(PDSP_mitragynine_Ki), mitragynine_pos, cex= PDSP_cex, pch=PDSP_pch1, col ='black')

#speciogynine
speciogynine_pos = 9
points(     df_alkaloids$lwr[2],   speciogynine_pos, pch = '|', cex=conf_cex)
points(     df_alkaloids$upr[2],   speciogynine_pos, pch = '|', cex=conf_cex)
segments(x0=df_alkaloids$lwr[2],y0=speciogynine_pos,x1=df_alkaloids$upr[2],y1=speciogynine_pos)
points(log10(PDSP_speciogynine_Ki), speciogynine_pos, cex=PDSP_cex, pch=PDSP_pch1, col= 'forestgreen')

#7oh-mitragynine
oh7_pos = 8
points(     df_alkaloids$lwr[7],   oh7_pos, pch = '|', cex=conf_cex)
points(     df_alkaloids$upr[7],   oh7_pos, pch = '|', cex=conf_cex)
segments(x0=df_alkaloids$lwr[7],y0=oh7_pos,x1=df_alkaloids$upr[7],y1=oh7_pos)
points(log10(PDSP_7oh_mitragyinine_Ki), oh7_pos, cex=PDSP_cex, pch=PDSP_pch1, col ='forestgreen')

#-----------------------------------------------------

#ajmalicine
ajma_pos = 7
points(     df_alkaloids$lwr[8],   ajma_pos, pch = '|', cex=conf_cex)
points(     df_alkaloids$upr[8],   ajma_pos, pch = '|', cex=conf_cex)
segments(x0=df_alkaloids$lwr[8],y0=ajma_pos,x1=df_alkaloids$upr[8],y1=ajma_pos)
points(log10(PDSP_ajmalicine_Ki), ajma_pos, cex=PDSP_cex, pch=PDSP_pch1, col= 'forestgreen')

#tetrahydroalstonine
tetra_pos = 6
points(     df_alkaloids$lwr[9],   tetra_pos, pch = '|', cex=conf_cex)
points(     df_alkaloids$upr[9],   tetra_pos, pch = '|', cex=conf_cex)
segments(x0=df_alkaloids$lwr[9],y0=tetra_pos,x1=df_alkaloids$upr[9],y1=tetra_pos)
points(log10(PDSP_tetrahydroalstonine_Ki), tetra_pos, cex=PDSP_cex, pch='<', col= 'purple')

#-----------------------------------------------------

#corynoxine_b
coryB_pos = 5
points(     df_alkaloids$lwr[11],   coryB_pos, pch = '|', cex=conf_cex)
points(     df_alkaloids$upr[11],   coryB_pos, pch = '|', cex=conf_cex)
segments(x0=df_alkaloids$lwr[11],y0=coryB_pos,x1=df_alkaloids$upr[11],y1=coryB_pos)
points(log10(PDSP_corynoxine_b_Ki), coryB_pos, cex=PDSP_cex, pch=PDSP_pch1, col= 'forestgreen')

#isorhynchophylline
isorhyncho_pos = 4
points(     df_alkaloids$lwr[13],   isorhyncho_pos, pch = '|', cex=conf_cex)
points(     df_alkaloids$upr[13],   isorhyncho_pos, pch = '|', cex=conf_cex)
segments(x0=df_alkaloids$lwr[13],y0=isorhyncho_pos,x1=df_alkaloids$upr[13],y1=isorhyncho_pos)
points(log10(PDSP_isorhynchophylline_Ki), isorhyncho_pos, cex=PDSP_cex, pch=PDSP_pch1, col= 'forestgreen')

#corynoxeine
corynoxeine_pos = 3
points(     df_alkaloids$lwr[17],   corynoxeine_pos, pch = '|', cex=conf_cex)
points(     df_alkaloids$upr[17],   corynoxeine_pos, pch = '|', cex=conf_cex)
segments(x0=df_alkaloids$lwr[17],y0=corynoxeine_pos,x1=df_alkaloids$upr[17],y1=corynoxeine_pos)
points(log10(PDSP_corynoxeine_Ki), corynoxeine_pos, cex=PDSP_cex, pch='<', col= 'purple')

#isocorynoxeine
isocorynoxeine_pos = 2
points(     df_alkaloids$lwr[18],   isocorynoxeine_pos, pch = '|', cex=conf_cex)
points(     df_alkaloids$upr[18],   isocorynoxeine_pos, pch = '|', cex=conf_cex)
segments(x0=df_alkaloids$lwr[18],y0=isocorynoxeine_pos,x1=df_alkaloids$upr[18],y1=isocorynoxeine_pos)

points(log10(PDSP_isocorynoxeine_Ki), isocorynoxeine_pos, cex=PDSP_cex, pch='<', col= 'purple')

axis(1, at=-2:7, las = 1)
axis(2, at=2:12, las = 1, labels=c('Isocorynoxeine'    , 'Corynoxeine', 
                                   'Isorhynchophylline', 'Corynoxine B',
                                   'Tetrahydroalsonine', 'Ajmalicine' , 
                                   '7OH-mitragynine'   , 'Speciogynine', 
                                   'Mitragynine'       , 'Fentanyl'   , 
                                   'Carfentanil') )
```

S3 Fig (Main text Figure 3A): Binding affinity predictions using the mu opioid regression model. The model predictivity interval is shown by the error bars, while the experimentally determined binding affinities are presented as symbols in panel A. The measured binding affinities of fentanyl and carfentanil are presented, for comparison, as the black squares and the normalized mitragynine binding affinity is presented as the black diamond. Panel A also presents the measured binding affinities of the remaining eight kratom alkaloids. The green diamonds indicate the experimentally measured binding affinities, while the purple greater than symbolindicates that no binding occurred below 10 µM.

```
par(mar=c(5,8,4,2))
plot(log10(PDSP_7oh_mitragyinine_Ki), 15, ylim=c(-2.5, 12.5), xlim=c(-2,7), ylab=''
     , xlab='log(Ki) [nM]', cex=2, pch=18, col ='white', yaxt='n')

#-----------------------------------------------------

#carfentanil
points(model_info$model_logKi[6], car_pos, cex=fent_cex, pch=fent_pch, col ='black')

#fentanyl
points(model_info$model_logKi[3], fent_pos, cex=fent_cex, pch=fent_pch, col ='black')

#-----------------------------------------------------

#speciociliatine 
speciociliatine_pos = 10
points(     df_alkaloids$lwr[3],   speciociliatine_pos, pch = '|', cex=conf_cex)
points(     df_alkaloids$upr[3],   speciociliatine_pos, pch = '|', cex=conf_cex)
segments(x0=df_alkaloids$lwr[3],y0=speciociliatine_pos,x1=df_alkaloids$upr[3],y1=speciociliatine_pos)
#points(log10(233), speciociliatine_pos, cex=PDSP_cex, pch=PDSP_pch1, col = 'forestgreen')

#mitraciliatine 
mitraciliatine_pos =  9
points(     df_alkaloids$lwr[4],   mitraciliatine_pos, pch = '|', cex=conf_cex)
points(     df_alkaloids$upr[4],   mitraciliatine_pos, pch = '|', cex=conf_cex)
segments(x0=df_alkaloids$lwr[4],y0=mitraciliatine_pos,x1=df_alkaloids$upr[4],y1=mitraciliatine_pos)


#paynantheine
paynantheine_pos = 8
points(     df_alkaloids$lwr[5],   paynantheine_pos, pch = '|', cex=conf_cex)
points(     df_alkaloids$upr[5],   paynantheine_pos, pch = '|', cex=conf_cex)
segments(x0=df_alkaloids$lwr[5],y0=paynantheine_pos,x1=df_alkaloids$upr[5],y1=paynantheine_pos)

#corynanthedine 
corynanthedine_pos =  7
points(     df_alkaloids$lwr[6],   corynanthedine_pos, pch = '|', cex=conf_cex)
points(     df_alkaloids$upr[6],   corynanthedine_pos, pch = '|', cex=conf_cex)
segments(x0=df_alkaloids$lwr[6],y0=corynanthedine_pos,x1=df_alkaloids$upr[6],y1=corynanthedine_pos)

#corynoxine 
corynoxine_pos =  6
points(     df_alkaloids$lwr[10],   corynoxine_pos, pch = '|', cex=conf_cex)
points(     df_alkaloids$upr[10],   corynoxine_pos, pch = '|', cex=conf_cex)
segments(x0=df_alkaloids$lwr[10],y0=corynoxine_pos,x1=df_alkaloids$upr[10],y1=corynoxine_pos)

#rhynchophylline 
rhyncho_pos =  5
points(     df_alkaloids$lwr[13],   rhyncho_pos, pch = '|', cex=conf_cex)
points(     df_alkaloids$upr[13],   rhyncho_pos, pch = '|', cex=conf_cex)
segments(x0=df_alkaloids$lwr[13],y0=rhyncho_pos,x1=df_alkaloids$upr[13],y1=rhyncho_pos)

#-----------------------------------------------------

#speciofoline 
speciofoline_pos =  4
points(     df_alkaloids$lwr[14],   speciofoline_pos, pch = '|', cex=conf_cex)
points(     df_alkaloids$upr[14],   speciofoline_pos, pch = '|', cex=conf_cex)
segments(x0=df_alkaloids$lwr[14],y0=speciofoline_pos,x1=df_alkaloids$upr[14],y1=speciofoline_pos)

#isospeciofoline 
isospeciofoline_pos =  3
points(     df_alkaloids$lwr[15],   isospeciofoline_pos, pch = '|', cex=conf_cex)
points(     df_alkaloids$upr[15],   isospeciofoline_pos, pch = '|', cex=conf_cex)
segments(x0=df_alkaloids$lwr[15],y0=isospeciofoline_pos,x1=df_alkaloids$upr[15],y1=isospeciofoline_pos)

#mitrafoline 
mitrafoline_pos =  2
points(     df_alkaloids$lwr[16],   mitrafoline_pos, pch = '|', cex=conf_cex)
points(     df_alkaloids$upr[16],   mitrafoline_pos, pch = '|', cex=conf_cex)
segments(x0=df_alkaloids$lwr[16],y0=mitrafoline_pos,x1=df_alkaloids$upr[16],y1=mitrafoline_pos)

#mitraphylline 
mitraphylline_pos =  1
points(     df_alkaloids$lwr[19],   mitraphylline_pos, pch = '|', cex=conf_cex)
points(     df_alkaloids$upr[19],   mitraphylline_pos, pch = '|', cex=conf_cex)
segments(x0=df_alkaloids$lwr[19],y0=mitraphylline_pos,x1=df_alkaloids$upr[19],y1=mitraphylline_pos)

#isomitraphylline 
isomitraphylline_pos =  0
points(     df_alkaloids$lwr[20],   isomitraphylline_pos, pch = '|', cex=conf_cex)
points(     df_alkaloids$upr[20],   isomitraphylline_pos, pch = '|', cex=conf_cex)
segments(x0=df_alkaloids$lwr[20],y0=isomitraphylline_pos,x1=df_alkaloids$upr[20],y1=isomitraphylline_pos)

#speciophylline 
speciophylline_pos =  -1
points(     df_alkaloids$lwr[21],   speciophylline_pos, pch = '|', cex=conf_cex)
points(     df_alkaloids$upr[21],   speciophylline_pos, pch = '|', cex=conf_cex)
segments(x0=df_alkaloids$lwr[21],y0=speciophylline_pos,x1=df_alkaloids$upr[21],y1=speciophylline_pos)

#isospeciophylline 
isospeciophylline_pos =  -2
points(     df_alkaloids$lwr[22],   isospeciophylline_pos, pch = '|', cex=conf_cex)
points(     df_alkaloids$upr[22],   isospeciophylline_pos, pch = '|', cex=conf_cex)
segments(x0=df_alkaloids$lwr[22],y0=isospeciophylline_pos,x1=df_alkaloids$upr[22],y1=isospeciophylline_pos)

axis(1, at=-2:7, las = 1)
axis(2, at=-2:12, las = 1, labels=c( 'Isospeciophylline'  , 'Speciophylline',
                                    'Isomitraphylline'   , 'Mitraphylline',
                                    'Mitrafoline'        , 'Isospeciofoline',
                                    'Speciofoline'       , 'Rhycholphylline',
                                    'Corynoxine'         , 'Corynantheidine',
                                    'Paynantheine',
                                    'Mitraciliatine'     , 'Speciocilitine',
                                    'Fentanyl'           , 'Carfentanil') )
```

S4 Fig (Main text Figure 3B): Binding affinity predictions using the mu opioid regression model. The model predictivity interval is shown by the error bars. The measured binding affinities of fentanyl and carfentanil are presented, for comparison, as the black squares.

# Quality Control Key

Table S3: Quality control key for experimentally characterized alkaloids.

| ID | Name | Registration ID |
| --- | --- | --- |
| 1 | Mitragynine | NCGC00488797-01 |
| 2 | Speciogynine | NCGC00488796-01 |
| 7 | 7-hydroxymitragynine | NCGC00488460-01 |
| 11 | Ajmalicine | NCGC00163632-01 |
| 12 | Tetrahydroalstonine | NCGC00163632-02 |
| 14 | Corynoxine B | NCGC00482767-02 |
| 16 | Isorhynchophylline | NCGC00482766-02 |
| 20 | Corynoxeine | NCGC00482762-02 |
| 21 | Isocorynoxeine | NCGC00482763-01 |

# Experimental conditions

The binding studies were performed by the National Institute of Mental Health Psychoactive Drug Screening Program (NIMH PDSP), as described previously [Besnard et al Nature 2012 492, 215]. Complete details of the binding assay conditions and protocols are provided in the NIMH PDSP Assay protocol book [Roth 2018]. The following tables provide a summary of the experimental conditions.

Table S4: Cell culture conditions. Fetal bovine serum (FBS).

| Cell Line | Media | FBS | Temperature (\(^\circ\)C) | Antibiotic |
| --- | --- | --- | --- | --- |
| HEK | Dulbecco’s Modified Eagle’s Medium | 10 % | 37 | none |
| MDCK | Eagle’s Minimal Essential Media | 10 % | 37 | none |
| CHO | F12-K | 10 % | 37 | none |

Table S5: Radioligands, concentrations, and conditions used for the opioid (mu, kappa, delta), adrenergic (alpha-2A, 2B, 2C), and serotonin (5-HT1A and 5-HT2A) receptor binding assays.

| Receptor | Parental cells | Radioligand pKd \(\pm\) SEM (Kd, nM) | Radioligand used (nM) | Reference Ligand pKi\(\pm\)SEM (Ki, nM) |
| --- | --- | --- | --- | --- |
| 5-HT1A | Stable CHO | [\(^3\)H]-Way100635, 9.30\(\pm\) 0.04 (0.50) | 0.5-1.0 | 8-OH-DPAT 9.18\(\pm\) 0.02 (0.66) |
| 5-HT2A | HEKT | [\(^3\)H]-Ketanserin, 8.92\(\pm\) 0.04 (1.20) | 1.2-2.4 | Clozapine 8.23\(\pm\) 0.01 (5.91) |
| delta | Stable HEK293 | [\(^3\)H]-DADLE, 8.57\(\pm\) 0.05 (2.69) | 1.0-2.0 | Naltrindole 9.30\(\pm\) 0.02 (0.50) |
| kappa | Stable HEK293 | [\(^3\)H]-U69593, 9.08\(\pm\) 0.04 (0.83) | 0.6-1.2 | Salvinorin A 8.53\(\pm\) 0.02 (2.98) |
| mu | Stable HEK293 | [\(^3\)H]-DAMGO, 8.92\(\pm\) 0.05 (1.20) | 1.0-2.0 | DAMGO 8.75\(\pm\) 0.02 (1.76) |
| Alpha2A | Stable MDCK | [\(^3\)H]-Rauwolscine 8.46\(\pm\) 0.16 (3.47) | 1.0-3.0 | Oxymetazoline 8.35\(\pm\) 0.02 (4.51) |
| Alpha2B | HEK293T | [\(^3\)H]-Rauwolscine 8.74\(\pm\) 0.13 (1.81) | 1.5-2.0 | Yohimbine 8.24\(\pm\) 0.02 (2.81) |
| Alpha2C | Stable MDCK | [\(^3\)H]-Rauwolscine 9.02\(\pm\) 0.10 (0.96) | 0.5-1.0 | Oxymetazoline 7.38\(\pm\) 0.02 (41.6) |

Table S6: Experimental conditions for the opioid (mu, kappa, delta), adrenergic (alpha-2A, 2B, 2C), and serotonin (5-HT1A and 5-HT2A) receptor binding assays.

| Receptor | Conditions |
| --- | --- |
| serotonin (5-HT1A, 5-HT2A) | Standard Binding Buffer: 50 mM Tris HCl, 10 mM MgCl2, 0.1 mM EDTA, pH 7.4, RT Standard Wash Buffer: 50 mM Tris HCl, pH 7.4, cold |
| Opioid (delta, kappa, mu) | Standard Binding Buffer: 50 mM Tris HCl, 10 mM MgCl2, 0.1 mM EDTA, pH 7.4, RT Standard Wash Buffer: 50 mM Tris HCl, pH. 7.4, 4 C to 8 C |
| Adrenergic (Alpha-2A, 2B, 2C) | Alpha2 Binding Buffer: 50 mM Tris HCl, 5 mM MgCl2, pH 7.7, RT Standard Wash Buffer: 50 mM Tris HCl, pH 7.4, cold |
